# The potential of aptamers for the analysis of ceramic bound proteins found within pottery

**DOI:** 10.1038/s41598-024-70048-8

**Published:** 2024-08-27

**Authors:** Janez Kosel, Polonca Ropret

**Affiliations:** 1 Institute for the Protection of Cultural Heritage of Slovenia, Conservation Centre, Research Institute, Ljubljana, Slovenia; 2 Faculty of Chemistry and Chemical Technology, University of Ljubljana, Ljubljana, Slovenia

**Keywords:** Cultural heritage, Heritage science, Archaeology, Immunofluorescent microscopy, Confocal fluorescent microscopy, Aptamers, Antibodies, 3D topography scans, Immunochemistry, Biochemistry

## Abstract

Archaeological pottery are the most numerous objects found during excavations and reflect the culinary practices of the past. However, their functionality for cooking/storing specific foods or drinks cannot be deduced solely from comparing their shapes and sizes. Analysis of protein residues bound to ceramics can reveal the protein/animal type through their amino acid sequence, thus enabling direct identification of food types. Therefore, the aim of our experimental study was to test sixteen aptamers for the analysis of proteinaceous organic residues found within the porous structure of pottery. Traditionally prepared archaeological ceramic replicas were cooked for 5 days in various food/protein suspensions, were UV aged, buried for a year, excavated, and extensively cleaned. Their shards were analysed using immunofluorescence microscopy with aptamers. Results show that eight aptamers (*Clone1* and *Kirby* for egg residuals; *seqU5* and *BLG14* for milk residuals; *HA* for blood residuals; *Gli4* for gluten residuals; *Par1* for fish residuals; and *D1* for collagen residuals) produced a successful/specific immunofluorescence microscopy result when they were hybridised to shards containing target protein residuals. Interestingly, on whole egg control samples, when the egg lysozyme-targeting aptamer *Kirby* was used, fluorescence intensity was 3.1 times greater compared to that observed with anti-ovalbumin antibodies.

## Introduction

Food-related activities occur on a daily basis, so their social importance is constantly reinforced. Archaeological interest in the sociality of food has grown dramatically over the last decade, with diverse investigations into how culinary practices articulate with economics, politics, ethnicity, gender, ideology, status and religion. This provides for a detailed and complex reconstruction of past social lives in a wide variety of cultures and eras. The ubiquity of food in culture, and of its associated material culture in archaeological contexts, means that still more studies can and should be done. Archaeological pottery are the most numerous objects found during excavations and reflect the culinary practices of past cultures dating back to the 'Neolithic Revolution,' at which the link between the advent of pottery production and the shift to agriculture can be argued^1–3^. For example, Hendy et al.^4^ identified dietary proteins in 8000 years old pottery artefacts from the West Mound of the key early farming site of Çatalhöyük in Anatolia, revealing that this community processed mixes of cereals, pulses, dairy and meat products, and that particular vessels may have been reserved for specialized foods. Interestingly, the earliest known potters lived in eastern Asia long before the development of agriculture (20,000–12,000 cal BP) or a settled way of life^1^.

However, the greatest challenge facing pottery analysts is how to infer vessel function from form^5^. In other words, the shapes of pots can be misleading and frequently results in wrong designations of specific food types. Therefore, the relationship between the vessel’s shape and function is more of an archaeological fiction, unless the vessels are strictly designed for liquids—jugs, amphorae, etc. Moreover, for the smaller and lesser-known social groups, the lack of written accounts and knowledge of their socioeconomic lifestyle add to a wrong designation of function for specific food types (for example pottery which is not Roman in origin)^6^.

Excavated ceramic vessels or shreds of vessels may be targeted and biodeterioraed by xerophilic molds, which tolerate arid environments^7^. These secrete extracellular enzymes, which can degrade organic residuals within the porous ceramic structure, resulting in the loss of valuable biological information, which could otherwise be used for the identification of food ingredients and their biological source. Therefore, institutions are reformulating conservation principles to prevent the loss or degradation of biomolecular compounds, which had not been considered before due to the unavailability of new techniques and analysis methods.

Extraction of lipid constituents either after grinding a sherd of pottery or by simply extracting this matter with an organic solvent and the application of GC–MS analysis^8^ reveals the use of various commodities, either from animal or plant origin and can highlight the production of dairy products as early as the Neolithic period, giving evidence for the use of secondary products from breeding^9^. Moreover, the identification of plant oils gives new light on the knowledge that prehistoric people had of their natural environment^10^. For example, the analysis of pottery found in Bedik Country, Senegal^11^, or that used by the earliest farming communities of the European Atlantic seaboard^12^, or that of the Southwestern Atlantic coast of Europe (Portugal, Spain and France)^13^, or the one used by the Corded Ware culture (Finnish prehistoric sites 4500 years ago)^14^, or ceramics found within the stratified site of Zamostje 2 in the forest zone of the Volga-Oka region (Early Neolithic—Middle Neolithic (MN))^15^. However, this technique can lack tissue specificity and is often unable to disentangle signatures resulting from the mixing of different food products. In contrast to lipids, protein analysis within the organic residuals of ancient pottery provides the opportunity for improved tissue resolution of foodstuffs prepared in ceramics^16^, mainly because they hold rich molecular information encoded within the amino acids describing not only the protein type but also its animal origin, directly leading to food type identification^17^. Moreover, the abundance and composition of proteins also influences preservation, with hydrophobic proteins surviving more readily than other proteins or ancient nucleic acid molecules (DNA)^18^.

ELISA (Enzyme-Linked Immunosorbent Assay) and immunofluorescence microscopy use target specific antibodies, which bind to individual protein targets^19^, and have been applied for the characterization and identification of ceramic bound ancient proteins^20–24^. Therefore, these two techniques only analyse the target proteins in question, and for each protein, a separate specific antibody and an overall labelling secondary antibody need to be prepared using skilled biochemical practices. 96-well plate VIS spectrophotometer (for ELISA) or a fluorescent microscope (for immunofluorescence microscopy) are also required. Even though immunological techniques are very specific, antibodies possess numerous disadvantages such as a complicated and up to a yearlong biological production with high batch-to-batch variability, highly insatiable protein structure which is easily denatured and commonly suffers from cross-reactivity problems due to nonspecific binding or loss of epitopes, a short life span regardless of freezing and irreversible loss of function after repeated thawing^25^. The large size of antibodies (≈150 kDa) can sterically limit their access to targets and their penetration into the sample^25^. Additionally, it is very difficult to induce an immune response, necessary for antibody production, by injecting proteins, which are highly conserved throughout evolution and contain numerous copies of uninterrupted amino-acid repeats. Unfortunately, storage proteins, which are usually the most preserved ancient proteins, contain large sections of such repeats.

Therefore, there is a need to search for alternative molecular recognition elements to antibodies and one promising option are aptamers. These are single-stranded oligonucleotides generated via chemical synthesis (Systematic Evolution of Ligands by Exponential enrichment or SELEX), which includes reiterative selection cycles, that bind their selected targets with high affinities and specificities, via folding into specific secondary ligand binding structures (their native form)^26^. The first selection cycle includes the introduction of a protein target into a molecular pool containing aptamers (≈50 nucleotide long oligomers) of all possible sequence combinations. After incubation the non-bonded aptamers are washed away and the bonded ones are released from targets using thermal denaturation. For the next cycle only the bonded aptamers are used in their native state and washing step is increased to only select the most specific and strongly bonded aptamers^25^. The final product constitutes promising alternatives to antibodies due to its high stability at room temperature and its long shelf-life (several years), no batch-to-batch variability and its reversible denaturation without loss of function. Aptamers have a low molecular weight (≈8 KDa) which gives them high surface density and excellent sample penetration. Chemical synthesis is inexpensive, fast (2–3 weeks), and produces a dense number of aptameric units (100 μg of aptamers equals 1 mg of antibodies). Strict selection with the tailored isolation process ensures their very high affinity and a greater specificity rate to antibodies^25^.

Therefore, the aim of our study was to by-pass the disadvantages of current antibody based methods by employing highly specific, stable and reliable aptamers for immunofluorescence microscopy instead of antibodies. The potential of aptamers for application was tested on cross-sections of resin embedded ceramic model samples and their effectiveness in immunofluorescence microscopy was compared with that of the antibodies. In this way, we aimed to demonstrate the feasibility of using aptamers for the analysis of proteinaceous organic residues found within the porous structure of historic pottery in future immunofluorescence microscopy and ELASA (Enzyme-Linked Aptamer Sorbent Assay) applications.

## Materials and methods

### Preparation of fresh control samples

Archaeological ceramic replicas (vessels) were traditionally manufactured and burned in a wood heap, by the Pottery Center Bahor (Institute for the Development and Promotion of Pottery 3,714,195,000, Šaleška cesta 1, 3320 Velenje; Slovenia). Traditional manufacture ensures that the final product is porous facilitating the retention of organic compounds within the ceramic matrix. This is because natural clays and minerals are used, which contain inherent porosity. Moreover, lower burning temperatures within a wood heap to those of modern furnaces contribute to its porosity^27^. Replicas were washed and cleaned three times with Milli-Q water and were broken into ceramic shards (~ 3 × 3 cm pieces) using a chisel and a hammer. Control samples were prepared on ceramic shards, and each shard was impregnated with either one of the two suspensions: (a) 50 g of whole chicken egg mixed with a stick blender; and (b) 5 g of lysozyme from chicken egg white (L6876-5G, Sigma Aldrich) and 50 g of MilliQ water (final lysozyme concentration of 100 g/L).

Suspensions were applied onto the concave surfaces of the ceramic shards in a thick layer with a brush (Utrecht Manglon 2630-B No. 10). The prepared samples were left to dry at room temperature, on covered plastic stands, for a period of 1 week. Importantly, these ceramic control samples were not treated with heat and were not aged. This was done to increase the likelihood of a successful binding of aptamers/antibodies to protein targets within these samples.

### Preparation of heated and aged model samples

Shards (prepared in the same manner as for the control samples) were placed in a glass beaker, which was then filled with 100 ml of one of the next suspensions: (a) 123 g of raw egg white and 110 g of MilliQ water; (b) 71 g of raw fish traut musscle tissue (without skin) and 123 g of MilliQ water mixed with a stick blender; (c) 30 g of Manitoba wheat flour '00' and 93 g of 70% ethanol (prepared with MilliQ water); (d) 5 g of gelatin from bovine skin (G6650, Sigma Aldrich) and 100 g of MilliQ water; (e) 58 g of raw beef and 110 g of MilliQ water mixed with a stick blender; (f) 57 g of locally produced black pudding (blood sausage made of pork) and 110 g of MilliQ water mixed with a stick blender; (g) 5 g of haemoglobin from bovine blood (08,449, Sigma Aldrich) and 50 g of MilliQ water; (h) 5 g of lysozyme from chicken egg white (L6876-5G, Sigma Aldrich) and 50 g of MilliQ water (final lysozyme concentration of 100 g/L); (i) locally produced raw milk (unpasteurized); and (j) 0.5 g of albumin from chicken egg white (ovalbumin) (A5378, Sigma Aldrich) and 5 g of MilliQ water.

To reduce evaporation, all glass beakers were covered with aluminium foil and cooked in an oven for 5 days at 85 °C, following the recommendations of Barker et al.^28^. Barker et al.^28^ state that during cooking in pottery, protein residues bind to clay surfaces in vessel walls through a variety of primarily non-covalent interactions. Each day, the beakers, containing the suspensions and the ceramic shards, were removed from the oven for a period of 30 min. During this time, their content was mixed for 30 min on a magnetic stirrer (50 rpm) and the evaporated water was replaced with Milli-Q water. After 5 days of cooking, clean shards were dried for one day and artificially aged using the Blak-Ray XX-15BLB UV Bench Lamps (USHIO, 15 W, 365 nm; 0.6 Amps; 95–0042-11; UVP LLS, USA) for 72 h. After artificial aging, shards were buried in peat soil (Poljanska cesta 40, 1000 Ljubljana; pH 5; less than 1% nitrogen, with phosphorus) for a year, following the recommendations of Craig and Collins^22^ for the preparation of environmentally exposed heated ceramic model samples. After excavation ceramic shards were cleaned with a paper cloth and rinsed 10 times with Milli-Q water. In this way we removed all visual traces of organic/soil residuals (see Supplementary Fig. 3).

### Preparation of cross-sections of samples in resin

Firstly, a ~ 2 × 2 mm large piece of sample was removed from the concave surface of a control/model sample, using a hammer and a sharp slotted screwdriver*.* Any piece containing organic residuals, visible under a stereomicroscope, was rejected*.* The selected piece was embed in copolymer resin Kristal PS (Samson Kamnik LLC, Slovenia), containing a 2% concentration (w/w) of catalysator Kristal PS (Methyl ethyl peroxide (MEK); Samson Kamnik LLC, Slovenia). The resin was polymerised for 5 days at room temperature. Hardened samples were then polished on the table top polishing equipment RotoPol-15 (Struers Westlake, OH), with six successively finer grades of Micro-mesh abrasive MD-Dac pads (320, 600, 800, 1200, 2400 and 4000 mesh, Struers Westlake, OH). Paraffin oil (Agrolit LLC, Slovenia) was used as a lubricant only with the first polishing pad (320 mesh) and for later polishing steps no lubricant was applied. Finally, paraffin oil was removed by shortly wiping (to.to paper sheets*,* Tosama LLC, Slovenia) the samples with petroleum ether (Sigma Aldrich, Germany), and by dry wiping. Then samples were fixed onto a microscope glass slide, using a Fimo polymer clay (Staedtler Mars GmbH & Co. KG, Germany), so that the cross sections were facing upwards and were completely aligned with the ground surface.

### Hybridization of antibodies to sample cross-sections

**Figure 1:**
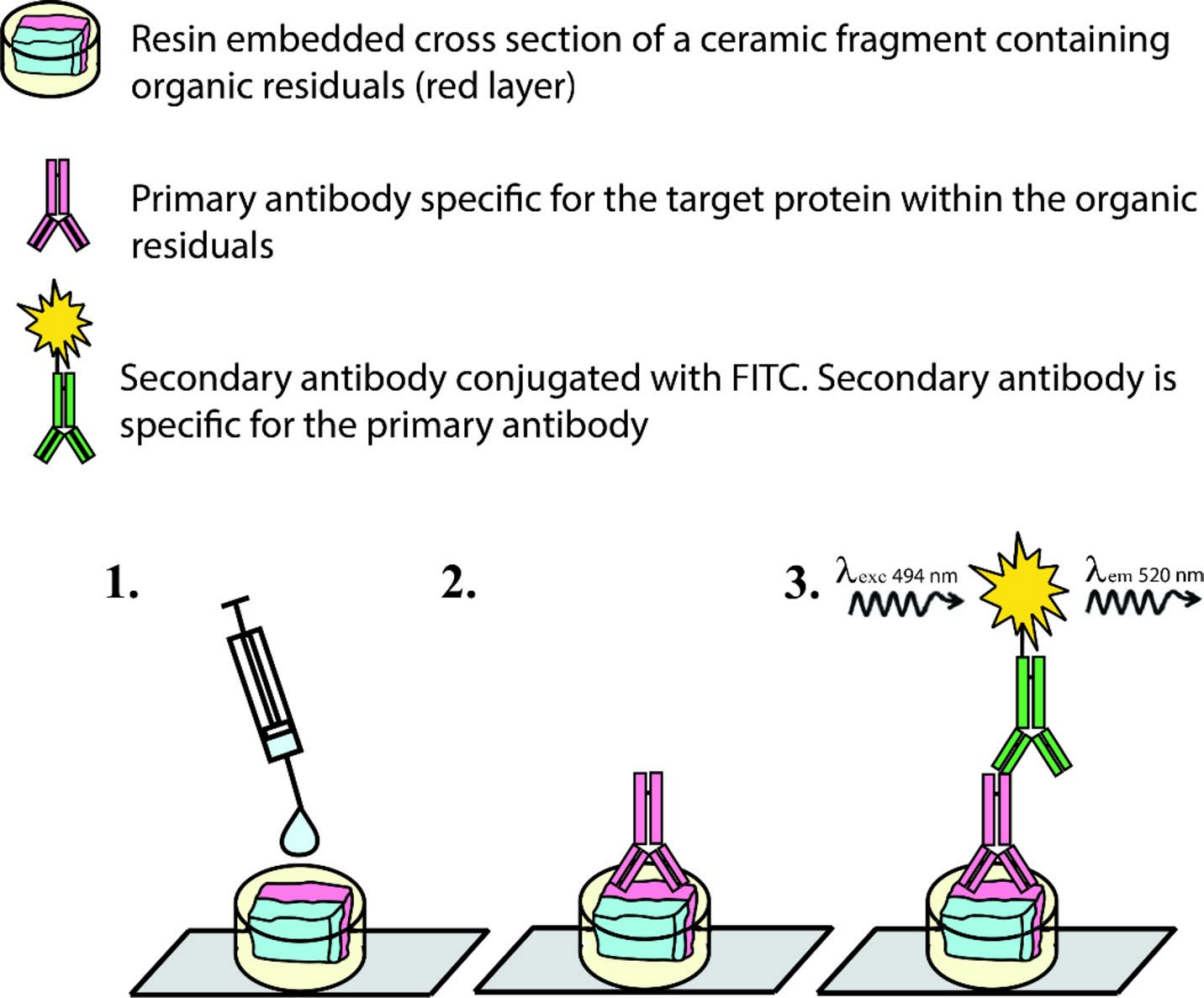
Schematic representation of hybridization of antibodies onto a sample cross-section for immunofluorescence microscopy (1. blocking solution application; 2. primary antibody application; and 3. secondary antibody application).

First, the exposed cross-section was covered with a 50 µl drop of 5% CSA (calf serum albumin; N4637, Sigma-Aldrich), which is used as a blocking solution (see Fig. 1). After a 60 min long incubation period at 23 °C, the blocking solution was removed using a pipette, and 50 µl of the primary anti-chicken egg albumin antibody (anti-ovalbumin Ab produced in rabbit, Sigma-Aldrich, C6534), diluted in blocking solution (1:10), was pipetted onto each cross-section and the samples were incubated overnight at 4 °C. Any unbound primary antibody was then washed off, by applying successive 100 µl drops of PBS at 4 °C to the sample, waiting 5 min, and removing the drop with a pipette. This washing process was repeated three times and great care was taken not to touch the sample itself with the tip of the pipette. After washing, 50 µl of the FITC (fluorescein isothiocyanate)-conjugated secondary anti-rabbit IgG (Ab produced in goat, Sigma-Aldrich, F0382), diluted in blocking solution (1:10), was pipetted onto each cross section and the samples were incubated for 2 h in the dark at room temperature. Unbound secondary antibodies were washed off, as described above, and during these and all of the subsequent steps, we removed all environmental sources of visible bright light. Finally, any remaining liquid around the sample was carefully removed, using a cotton swab (without touching the sample itself) and the samples were left to dry for 1 h in the dark.

### Aptamer selection and reconstruction

**Figure 2:**
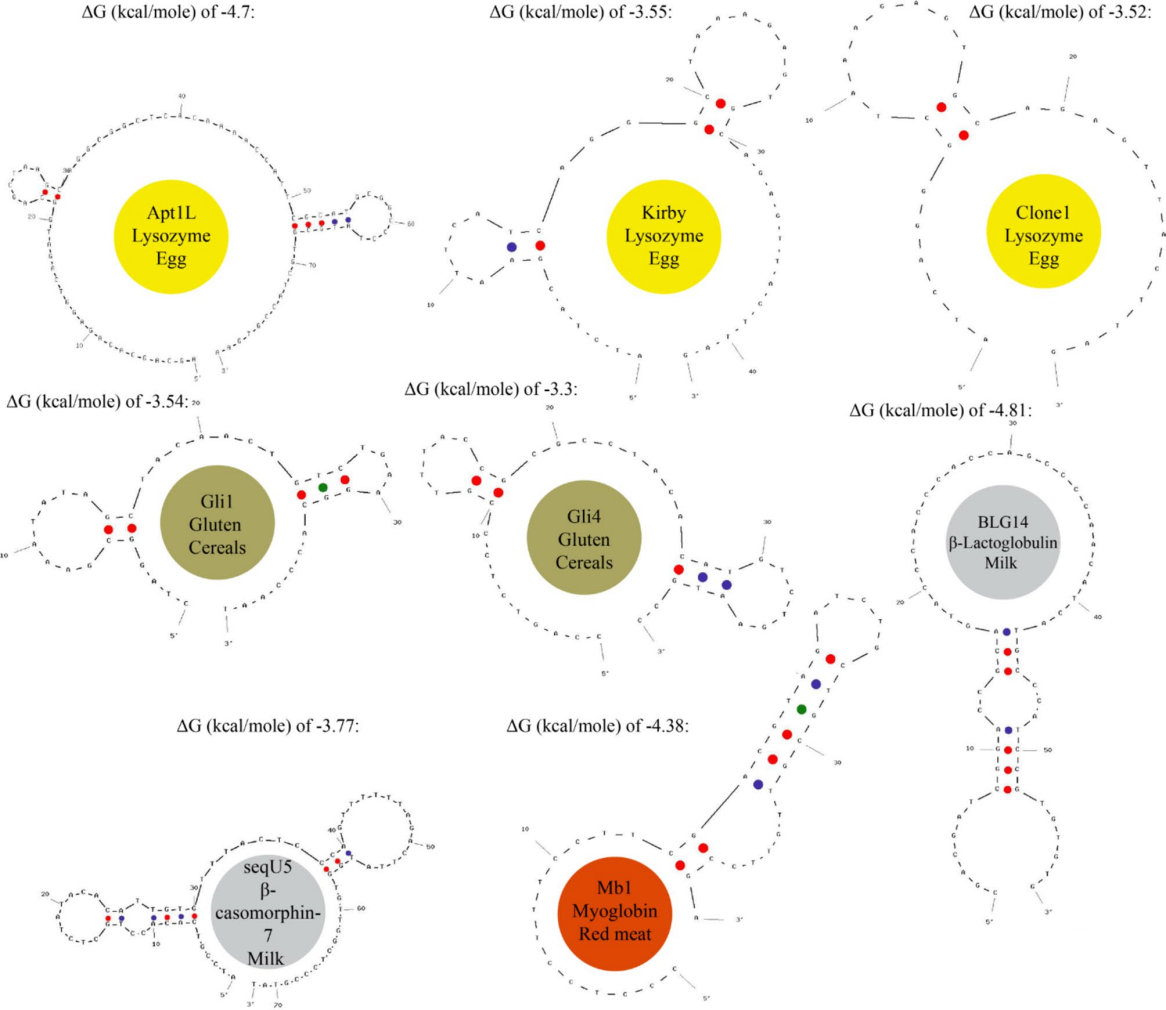
Folded secondary structures of aptamers, reconstructed within the OligoAnalyzer™ Online Tool, which target egg lysozyme; gluten (cereals); myoglobin (red meat) as well as β-casomorphin-7 and β-lactoglobulin within milk.

**Figure 3:**
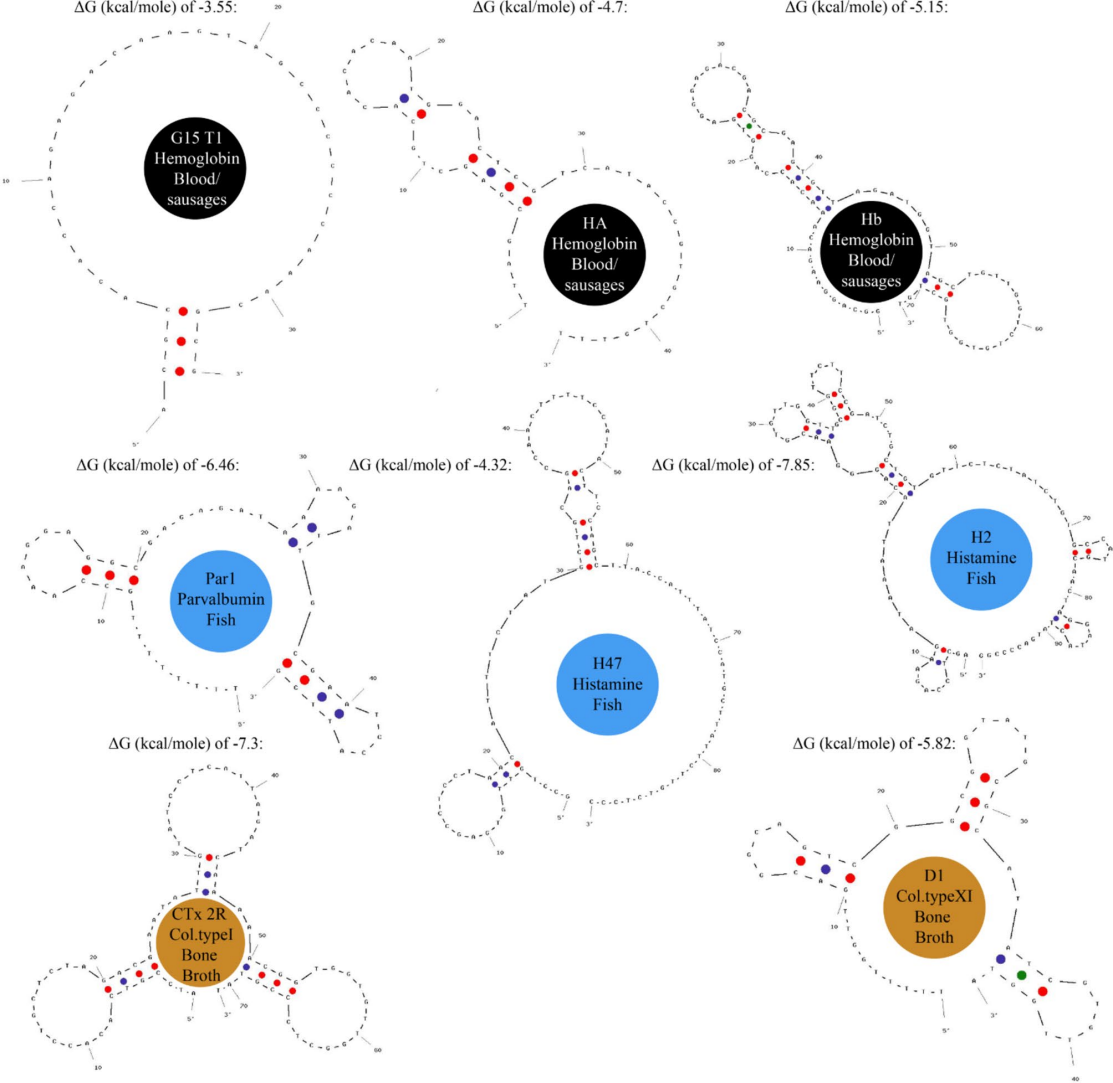
Folded secondary structures of aptamers, reconstructed within the OligoAnalyzer™ Online Tool, which target hemoglobin (black pudding); parvalbumin and histamine within fish; and collagen (bone broth).

Appropriate protein targets were listed according to LC–MS/MS proteomic studies of food residuals found in ancient ceramics^28–32^. The amino acid peptides found (along with histamine, which is not a peptide but is abundant in fish) were sequenced within these studies and thus any of the preserved proteins were identified. Then, within the published scientific articles, we searched for DNA aptamers, which specifically targeted the LC–MS/MS identified proteins (found within ancient ceramics), and for their single strand nucleic acid sequences (Table 1). In literature, these aptamers were developed via chemical synthesis (Systematic Evolution of Ligands by Exponential enrichment or SELEX), which includes reiterative selection cycles, that bind their selected protein targets with high affinities and specificities, via folding into specific secondary ligand binding structures^26^. The selected protein ligand targets are listed in Table 1 under the “target protein” column. According to the literature obtained DNA aptamer sequences, we used the OligoAnalyzer™ Online Tool (Integrated DNA Technologies, Inc.) with default values (Figs. 2 and 3) to reconstruct the folded (native) secondary structures of the aptamers. Within this software, the ΔG values of the reconstructed aptamers were calculated according to the next equation:

1 $$\Delta \text{G}= -\text{RT ln}\left(\frac{1}{\text{K}}\right),$$

where K is the equilibrium constant of the binding reaction; R is the gas constant with a value of 8.314 J K-1 mol-1; T is the temperature of the reaction [K]; and ΔG° is Gibbs free energy change under standard conditions [kcal mol^-1^]. A more negative ΔG indicates a stronger binding affinity of aptamers to target proteins (Figs. 2 and 3).

**Table 1:** Sixteen DNA sequences of aptamers employed within our study, for targeting proteins within eggs, cereals, milk, blood, red meat, fish and bone.

Diet	Target protein	Name	5'—3' end DNA single strand sequence modified with 5' end biotinylation [Btn]	T_m_ [°C]	Source of aptamer sequence in literature
Chicken egg	Heated (90 °C) chicken egg lysozyme from Sigma-Aldrich	*Clone1*	[Btn]ATCAGGGCTAAAGAGTGCAGAGTTACTTAG	66.1	^33^
		*Kirby*	[Btn]ATCTACGAATTCATCAGGGCTAAAGAGTGCAGAGTTACTTAG	74.5	^34^
		*Apt1L*	[Btn]AGCAGCACAGAGGTCAGATGGCAGCTAAGCAGGCGGCTCACA AAACCATTCGCATGCGGCCCTATGCGTGCTACCGTGAA	98.0	^35^
Cereals	Wheat gluten extract coocked at 50 °C for 40 min	*Gli 4*	[Btn]CCAGTCTCCCGTTTACCGCGCCTACACATGTCTGAATGCC	77.0	^36^
		*Gli 1*	[Btn]CTAGGCGAAATATAGCTACAACTGTCTGAAGGCACCCAAT	71.9	
Milk	Heated (90 °C) β-casomorphin-7 (β- casein A1) from TechnoConcept (India Pvt. Ltd.)	*seqU5*	[Btn]ATCCGTCACACCTGCTCTATACACATTGTGTTTACTCCCAGTTT TTTAGACTTATGGTGTTGGCTCCCGTAT	86.3	^37^
	Heated (90 °C) β-lactoglobulin from Sigma-Aldrich	*BLG14*	[Btn]CGACGATCGGACCGCAGTACCCACCCACCAGCCCCAACATCA TGCCCATCCGTGTGTG	98.1	^38^
Blood/sausages	Heated (70 °C) total hemoglobin from Monojo (Amman Jordan)	*G15 T1*	[Btn]ACGCACACCAGAGACAAGTAGCCCCCCAAACGCG	85.6	^39^
		*HA*	[Btn]TTAGCGAGCTGCACACACAATGGACTCGTCATACCGTGCTGTTT	85.8	^40^
		*Hb*	[Btn]GGCAGGAAGACAAACACCAGGTGAGGGAGACGACGCGAGTGTT AGATGGTAGCTGTTGGTCTGTGGTGCTGT	93.8	^41^
Red meat	Heated (70 °C) myoglobin from Sigma-Aldrich	*Mb1*	[Btn]CCCTCCTTTCCTTCGACGTAGATCTGCTGCGTTGTTCCGA	85.5	^42^
Fish	Boiled (for 30 min) fish parvalbumin extract	*Par1*	[Btn]TTTTTTTTGCCAAAGGAGGCGAGAGATAAAAGATTGCGAATCCATTCG	83.9	^43^
	Histamine	*H2*	[Btn]AGCTCCAGAAGATAAATTACAGGGAACGTGTTGGTTGCGGTTCTTCC GATCTGCTGTGTTCTCTATCTGTGCCATGCAACTAGGATACTATGACCCCGG	92.8	^44^
		*H47*	[Btn]GCCTGTTGTGAGCCTCCTAACATTTCTATGCTGCAGCCAACTTTTCCAT ACTTCCAGCTTACCATTTATCCATGCTTATTCTTGTCTCCC	89.7	^45^
Bone Broth	α1-chain of a human collagen type XI	*D1*	[Btn]TTTTTGGTTGACGGCAGTCGGCGGTATGCGCATATCGTGTTGGTA	76.1	^46^
	C-telopeptide of human type I collagen	*CTx 2R*	[Btn]ATCCGTCACACCTGCTCTAGACGAATATTGTATCCTCATTAGATCAAAAACGGGTGGTGTTGGCTCCCGTAT	56.0	^47^

### Aptamer synthesis and preparation

The collected aptamer sequences were sent to Sigma-Aldrich (Switzerland), where they were synthesised (synthesis scale of 0.2 µmol), HPLC purified and modified with 5' end biotinylation (Btn). In our laboratory, the delivered aptamers were briefly centrifuged which ensured us that the lyophilized aptamer pellet was at the bottom of the tube. Then they were resuspended in Aptamer Resuspension Buffer (Product # RTW0001; Base Pair Biotechnologies, Inc.) to achieve a 100 µM concentration (using microliter volumes indicated on the Sigma-Aldrich aptamer synthesis report). After that, they were incubated at room temperature for 30 min, vortexed for 20 s and centrifuged at 10,000 × g for 1 min. Aliquots were created and stored at –20 °C.

Prior to use, the working aptamer solution was prepared by thawing the aliquots and by diluting them (1:10) in the Aptamer Folding Buffer (Product # RTW0003; Base Pair Biotechnologies, Inc.). Finally, the solution was transferred into 200 µl PCR tubes (strips), heated within the MyCycler Thermal PCR Cycler (BioRad) to 93 °C for 5 min and incubated at room temperature for 25 min.

### Hybridization of aptamers to sample cross-sections

**Figure 4:**
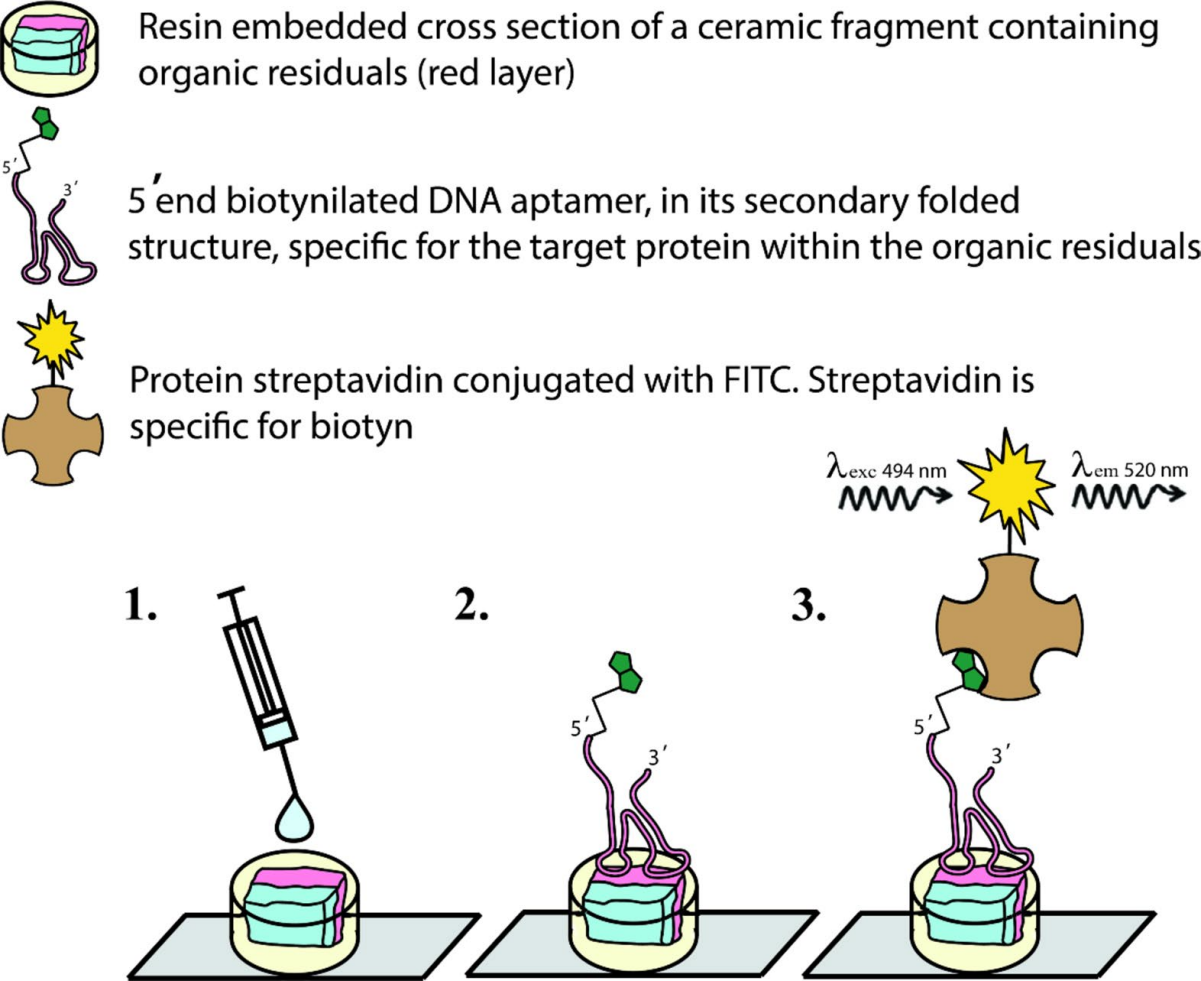
Schematic representation of hybridization of aptamers onto a sample cross-section for immunofluorescence microscopy (1. blocking solution application; 2. aptamer application; and 3. streptavidin application).

First, the exposed cross-section was covered with a 50 µl drop of 5% CSA (N4637, Sigma-Aldrich). After a 60 min long incubation period at 23 °C, the blocking solution was removed using a pipette, and 50 µl of the working aptamer solution, was pipetted onto each cross-section and the samples were incubated overnight at room temperature (see Fig. 4). Any unbound aptamer was then washed off, by applying successive 100 µl drops of aptamer washing buffer (PBS with 5 mM MgCl_2_) to the sample, waiting 5 min, and removing the drop with a pipette. This washing process was repeated three times. After washing, 50 µl of the FITC (fluorescein isothiocyanate)-conjugated streptavidin (S0966, TCI chemicals), diluted in aptamer washing buffer (1:33), was pipetted onto each cross-section and the samples were incubated for 2 h in the dark at room temperature. Unbound streptavidin was washed off, as described above, and during these and all of the subsequent steps, we removed all environmental sources of visible bright light. Finally, any remaining liquid around the sample was carefully removed, using a cotton swab (without touching the sample itself) and the samples were left to dry for 1 h in the dark.

### Microscopy

#### Fluorescent microscopy

Prior to and after the hybridization, the sample cross-sections were photographed in the widefield fluorescence observation mode of the Axio Imager.Z2m LSM 800 microscope (Carl Zeiss GmbH, Germany), running the software Zen Blue 2.5 (Carl Zeiss GmbH, Germany). The HXP unit (metal halide fluorescence light lamp module; 120 V) coupled with the cube filter set 10 (excitation: 450–490 nm, Beamsplitter: FT 510, emission: 515–565 nm; Carl Zeiss GmbH, Germany, 488,010–9901-000) was turned on and within the Acquisition tab of the Zen Blue 2.5 software, under the »widefield« screen (within the smart setup *menu*), the FITC channel (519 nm) was selected. The intensity of the lamp module was set to 20.0% and the shift value (under exposure time) was set to 80%. Images were captured at a magnification of 100 × using the Axiocam 503 camera in black and white mode and a green *pseudo colour (*false *colour*) was later added.

#### Fluorescence intensity calculation

**Figure 5:**
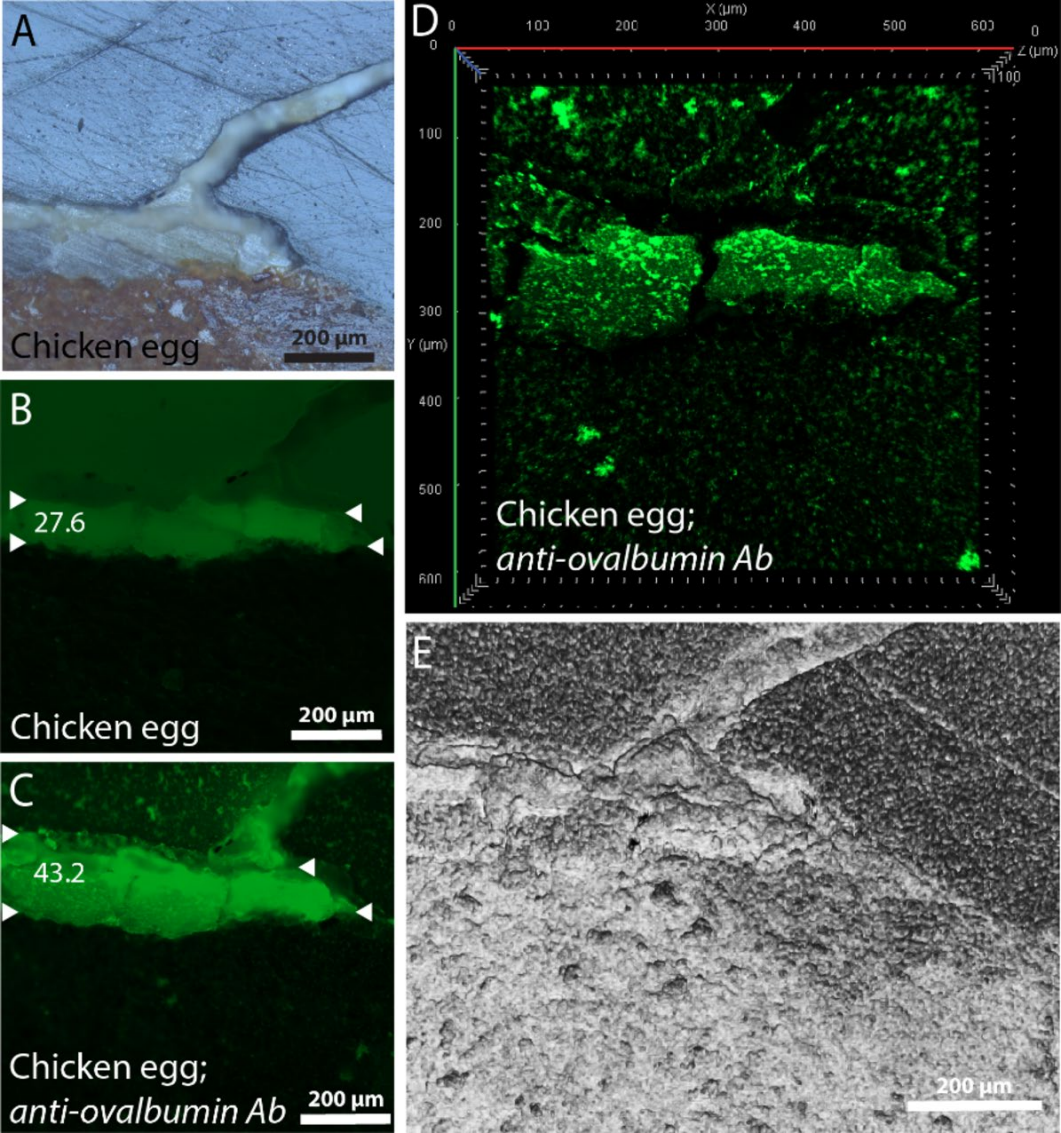
Cross-section images of the control sample, for which a chicken egg (whole egg) mix was directly applied onto the ceramic shard. It was photographed prior to anti-ovalbumin antibody (Ab) hybridisation using the reflected-light brightfield observation (frame A) and the widefield fluorescence observation (frame B) modes. After antibody hybridisation, the sample was again captured in the widefield observation mode under the same illumination conditions (frame C) and was laser scanned using confocal fluorescence microscopy (frame D) and surface topography scans (frame E). Fluorescence intensities of sample layers in frames B and C are marked with white, horizontally directed triangles.

**Figure 6:**
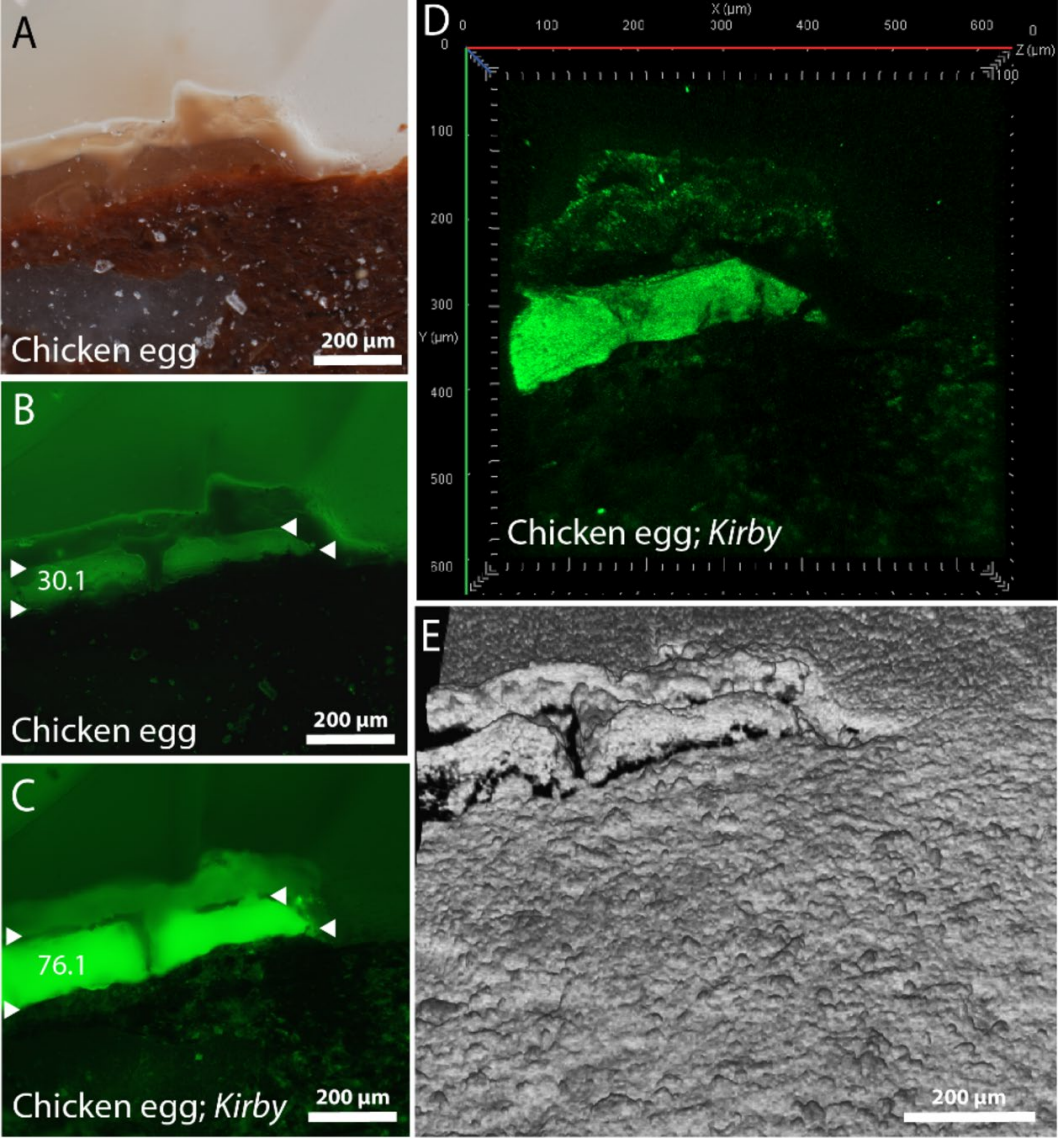
Cross-section images of the micro sample, for which a chicken egg (whole egg) mix was directly applied onto the ceramic shard. It was photographed, prior to hybridisation with the lysozyme (egg) targeting aptamer *Kirby*, using the reflected-light brightfield observation (frame A) and the widefield fluorescence observation (frame B) modes. After aptamer hybridisation, the sample was again captured in the widefield observation mode under the same illumination conditions (frame C) and was laser scanned using confocal fluorescence microscopy (frame D) and surface topography scans (frame E). Fluorescence intensities of sample layers in frames B and C are marked with white, horizontally directed triangles.

Fluorescence intensities (FI) were quantified from whole egg control samples images obtained using *widefield fluorescent* microscopy. Firstly, the .tif format was opened, within the ImageJ distribution Fiji, and the fluorescence intensity of the thick upper organic proteinous layer (defined by white triangles; see Figs. 5 and 6) was quantified, by marking the region of this layer using the »Polygon selections tool« and by selecting the »Measure« function (preselect »Area« and »Mean Gray Value« under the »Set measurements« options), within the »Analyse« menu.

#### Laser scanning confocal fluorescence microscopy

Hybridised sample cross sections were laser scanned by using the laser-scanning confocal *fluorescent* observation mode, within the Zeiss Axio Imager.Z2m LSM 800 microscope. Firstly, the LSM 800 laser module LM URGB (contains fibre-coupled, pigtailed and collimated lasers) was turned on and the anti-vibration table Vision IsoStation (Newport, USA), on which the microscope system is positioned, was filled with 1 bar of pressure using compressed air. The manual tube slider (for switching between VIS/Camera) was placed into the camera position (100% camera) and within the Acquisition tab of the Zen Blue 2.5 software, under the »LSM« screen (within the smart setup *menu*), the FITC channel (519 nm) was selected. The intensity of the 488 nm argon diode laser (10 mW, class 3B), used for the excitation of FITC, was set to 2%. Emission detection was via the LSM 800 MAT Confocal MA-detection module (Carl Zeiss GmbH, Germany), which consists of a main beam splitter (MBS), a variable pinhole with automatic alignment, two variable secondary dichroics (VSD) placed at 10 degree angle to incident beam for most effective excitation light suppression; and an emission filter in front of each of the two multi-alkali (MA) PMT confocal channel detectors. The pinhole was set to 1 Airy unit (AU) with an opening diameter of 36 µm. The digital gain of the PMT detectors was set to 0.9 (digital offset of 0), however the master gain settings (in V) varied from sample to sample. Finally, by defining the scanning boarders (set first/last), a 3D model (at 100 × magnification) was computer generated from a Z-stack of images, within an interval of 100 µm (each image was 2.6 µm apart).

#### Surface topography scans

The topography of hybridised cross section was laser scanned, by using the 405 nm *laser scanning* mode, within the above mentioned Zeiss microscope system. Therefore, the same physical units and microscope system settings were employed as in Sect. 3.8.3. Within the applications tab of the Zen Blue 2.5 software, under the »topography«, the image acquisition option was selected. The intensity of the 405 nm violet diode laser (5 mW, class 3B), used for scanning, was set to 10%. The pinhole was set to 1 Airy unit (AU) with an opening diameter of 25 µm. Even though the master gain settings varied from sample to sample, its values were always close to around 250 ± 10 V. In order to engulf the entire surface topography of a cross-section, the scanning boarders (set first/last) were set to acquire a 700 µm long interval of Z-stack images (each image was 0.41 µm apart; 100 × magnification) and these were saved as a CZI file format. This format was opened within the ImageJ distribution Fiji, and the images were reduced to 8-bits. Finally, by selecting the 3D Viewer option, within the »Plugins« menu, a 3D surface topography model was generated.

### Aptamer testing

Aptamers were tested using the ELISA procedure, according to the protocol published by Heginbotham et al.^48^ with some modifications. Firstly, protein suspensions (prepared in section "Immunofluporecence microscopy of control samples") were 50 fold diluted in sodium bicarbonate buffer (SBB: 100 mM of NaHCO3, pH 9.6), were separately applied onto Nunc-immuno microtiter plate (96 MicroWell Plates, MaxiSorp Sigma Aldrich; 80 μL/well of sample suspension/MilliQ water for negative control/bovine serum albumin A7906 (BSA; Sigma Aldrich; 10 μg/ml prepared in SBB) for standard), and were incubated overnight (2–8 °C). Afterwards, 300 μl of 5% CSA (N4637, Sigma-Aldrich) blocking solution was added, to prevent unspecific binding of antibodies in the further steps of the procedure. For the indirect ELISA, the working aptamer solution (prepared in Sect. 3.6) was diluted in Aptamer Folding Buffer, at a ratio of 1:10, and the streptavidin − alkaline phosphatase (AP) (S2890; Sigma Aldrich) was diluted in CSA, at a ratio of 1:100. For both aptamers and streptavidin − AP, 100 μl were applied to each well. To obtain enzyme–substrate reaction, 100 μl of colourless p-NPP (P7998, Sigma Aldrich) was added to the wells and incubated for 30 min at room temperature. The indicator was, in the case of a positive reaction, converted into a coloured product, by the reporting enzyme. Finally, the results were read using the Multiskan Sky (Thermo Scientific) spectrophotometer (optical density at 405 nm). Between each step, the wells were washed twice, using 300 μl of 0.02% Tween-20 in PBS, and twice with PBS only.

## Results

### Aptamer testing

Results of testing aptamers using the ELISA assay are presented in Supplementary Table 1. After the performed assay, aptamers for lysozyme (from chicken egg), when applied onto the lysozyme suspension wells, had OD_405nm_ values of 0.463 for *Clone1,* 0.380 for *Kirby* and 0.037 for *Apt1L.* Aptamers for gliadin (gluten), when applied onto the wheat flour suspension wells, had OD_405nm_ values of 0.577 for *Gli1* and 0.519 for *Gli4.* Aptamers for milk proteins, when applied onto the raw milk wells, had OD_405nm_ values of 0.510 for *seqU5* (casein) and 0.394 for *BLG14* (β-lactoglobulin)*.* Aptamers for haemoglobin, when applied onto the haemoglobin suspension wells, had OD_405nm_ values of 0.388 for *Hb*, 0.471 for *HA* and 0.015 for *G15 T1.* The aptamer *Mb1* (for myoglobin), when applied onto the raw beef suspension wells, had an OD_405nm_ value of 0.488. Aptamers for fish related compounds, when applied onto the fish traut suspension wells, had OD_405nm_ values of 0.412 for *Par1* (for parvalbumin), 0.087 for *H2* (for histamin) and 0.031 for *H47* (for histamin)*.* Finally, aptamers for collagen, when applied onto the gelatin suspension wells, had OD_405nm_ values of 0.400 for *D1* (collagen type XI) and 0.615 for *CTx 2R-2 h* (collagen type I)*.* For BSA standard and for negative control wells (MilliQ water), the signal was low (OD_405nm_ ˂ 0.1) for all tested aptamers.

### Immunofluporecence microscopy of control samples

To compare aptamer hybridisation results with the allready established antibody hybridisation procedure^49–51^, we hybiridsed the same whole egg control sample (chicken egg mix applied onto the ceramic shard), with either the lysozyme targeting aptamer *Kirby* or with the anti-ovalbumin antibody (see Figs. 5A and 6A). After both hybridization processes, fluorescence intensities (under the widefield fluorescence observation mode) in the thick upper whole egg layer increased from 28 to 43 (by 15) for the anti-ovalbumin antibody (Fig. 5B and C) and from 30 to 76 (by 46) for the aptamer Kirby (Fig. 6B and C). Therefore, in comparison with the use of antibodies, the increase in fluorescence intensity was 3.1 times greater for the Kirby aptamer. Moreover, the hybridisation with the aptamer produced a much clearer picture (Fig. 6C), and an even clearer localisation of fluorescence within the whole egg layer was observed in the confocal fluorescence observation *mode* (Fig. 6D). In contrast, antibody hybridisation produced smaller and larger fluorescent stains, mainly scattered around the polished resin area (Fig. 5C and D).

After both hybridisations (antibodies or aptamers), the upper whole egg layer swelled up and increased in size for about 1.5 times. The elevation of the egg layer, caused by the swelling, above the rest of the polished sample’s surface is clearly visible from the 3D topography models (Fig. 5E for the anti-ovalbumin Ab and Fig. 6E for the aptamer *Kirby*).

From the supplementary Figs. 1 K and 1L (yellow letters), we can observe that the negative control sample, with the thick layer of lysozyme applied to it, did not yield a successful immunofluorescence microscopy result after anti-ovalbumin antibody hybridization. This outcome is logical, as anti-ovalbumin antibodies exclusively target ovalbumin protein and not lysozyme.

### Immunofluorecence microscopy of model samples using aptamers

Model samples cooked in a suspension of egg white were hybridized with lysozyme targeting aptamers *Clone1, Kirby* and *Apt1L*; those cooked in lysozyme (extracted from egg white) or ovalbumin suspension were hybridized with *Clone1* and *Apt1L*; those cooked in raw milk were hybridized with *seqU5* (targeting casein) and *BLG14* (targeting β-lactoglobulin)*;* those cooked in a suspension of black pudding or haemoglobin were hybridized with haemoglobin targeting aptamers *G15 T1*, *HA* and *Hb*; those cooked in a suspension of wheat flour were hybridized with gliadin (gluten) targeting aptamers *Gli4* and *Gli1*; those cooked in a suspension of fish traut were hybridized with *Par1* (targeting parvalbumin), *H2* (targeting histamin) and *H47* (targeting histamin); those cooked in a suspension of gelatin (bovine skin) were hybridized with collagen targeting aptamers *D1* and *CTx 2R-2 h*; and finally those cooked in a suspension of beef were hybridized with a myoglobin targeting aptamer *Mb1*.

After analysis, immunofluorescence microscopy was not successful in highlighting target proteins in gelatin model sample hybridised with *CTx 2R-2 h*; in egg white and lysozyme model samples hybridised with *Apt1L*; in ovalbumin model samples hybridised with *Clone1* and *Apt1L* (Supplementary Fig. 1); in wheat flour model sample hybridised with *Gli1*; in traut fish model samples hybridised with *H2* and *H47*; in beef model sample hybridised with *Mb1*; and in black pudding and haemoglobin model samples hybridised with *G15 T1* and *Hb* (Supplementary Fig. 2)*.* The negative results, which were obtained when ovalbumin model samples (made from pure ovalbumin standard) were hybridised with *Clone1* and *Apt1L*, were expected, because these two aptamers only target the lysozyme protein and not ovalbumin.

Immunofluorescence microscopy was successful in the next cases: egg white model samples hybridised with *Clone1* and *Kirby*; lysozyme model sample hybridised with *Clone1*; raw milk model samples hybridised with *seqU5* and *BLG14*; black pudding and haemoglobin model samples hybridised with *HA*; wheat flour model sample hybridised with *Gli4*; fish traut model sample hybridised with *Par1*; and finally gelatin model sample hybridised with *D1*. In the below text, these successful cases are presented in detail.

**Figure 7:**
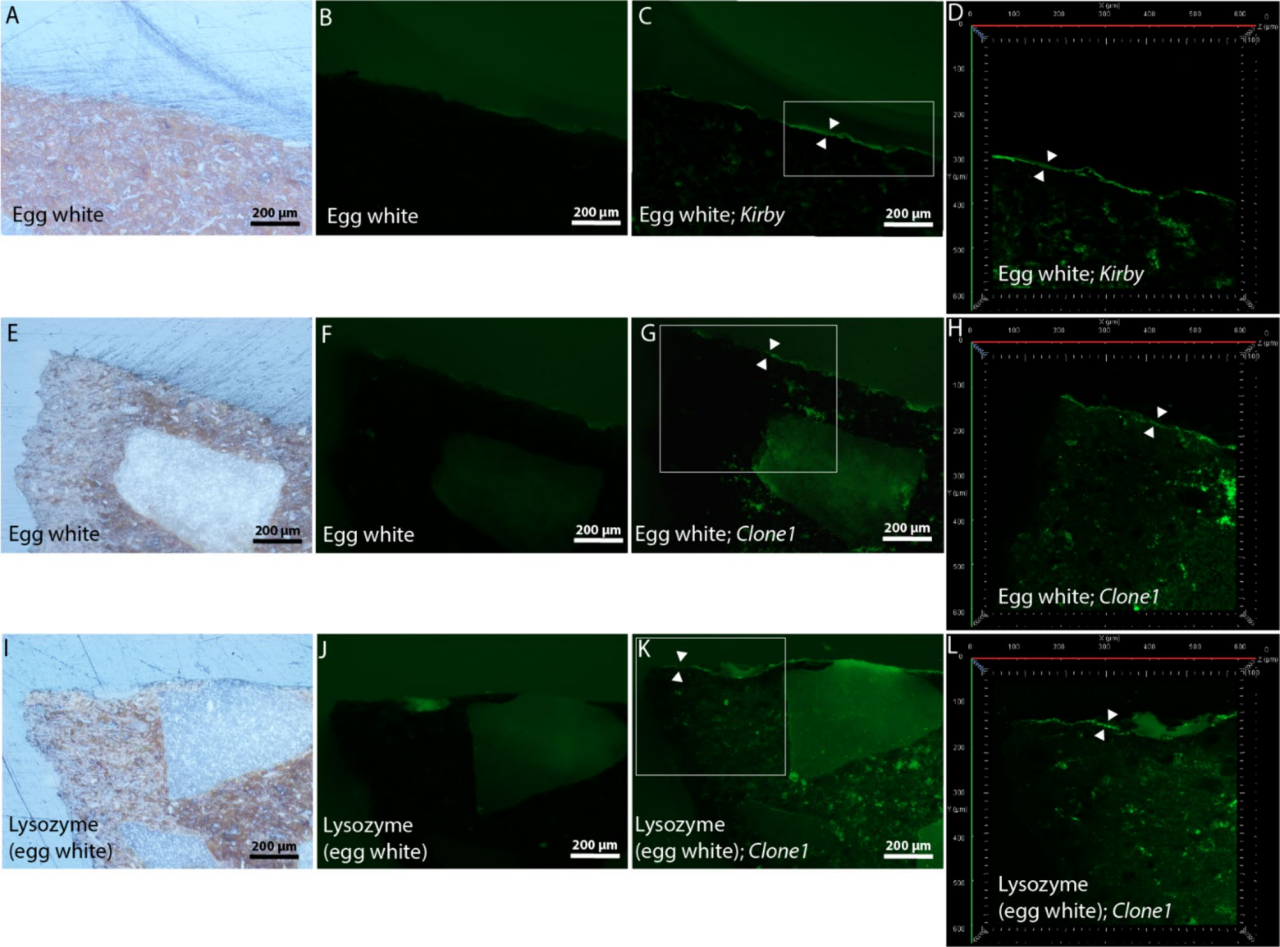
Cross-section images of model samples, which were cooked in a suspension of egg white or lysozyme (extracted from egg white). They were photographed, prior to hybridisation with lysozyme targeting aptamers *Kirby* (frames C and D) or *Clone1* (frames G, H, K and L), using the reflected-light brightfield observation (frames A, E and I) and the widefield fluorescence observation (frames B, F and J) modes. After aptamer hybridisations, samples were again captured in the widefield fluorescence observation mode under the same illumination conditions (frames C, G and K) and were laser scanned using confocal fluorescence microscopy (frames D, H and L). Upper layers of ceramic, which were directly exposed to cooking, are marked with white triangles.

Prior to hybridisation, the organic residuals, present within the thin most upper layer of ceramic, which was directly exposed to cooking in a suspension of egg white or lysozyme (Fig. 7A, E and I), exhibited a very weak autofluorescence (Fig. 7B, F and J). After the hybridisation of egg white or lysozyme samples with aptamers (*Kirby* or *Clone1* on egg white; *Clone1* on lysozyme), this layer (defined by white triangles in Fig. 7C, G and K) was highlighted with additional fluorescence, which enhanced its brightness under the widefield fluorescence observation mode (this applies to egg white as well as to lysozyme model samples). These enhancements were stronger and even clearer in the confocal fluorescence observation *mode, which excluded autofluorescence from microscopically small stones within the ceramic (*Fig. 7H*) as well as from the resin’s surface *reflection (Fig. 7D, H and L*).*

**Figure 8:**
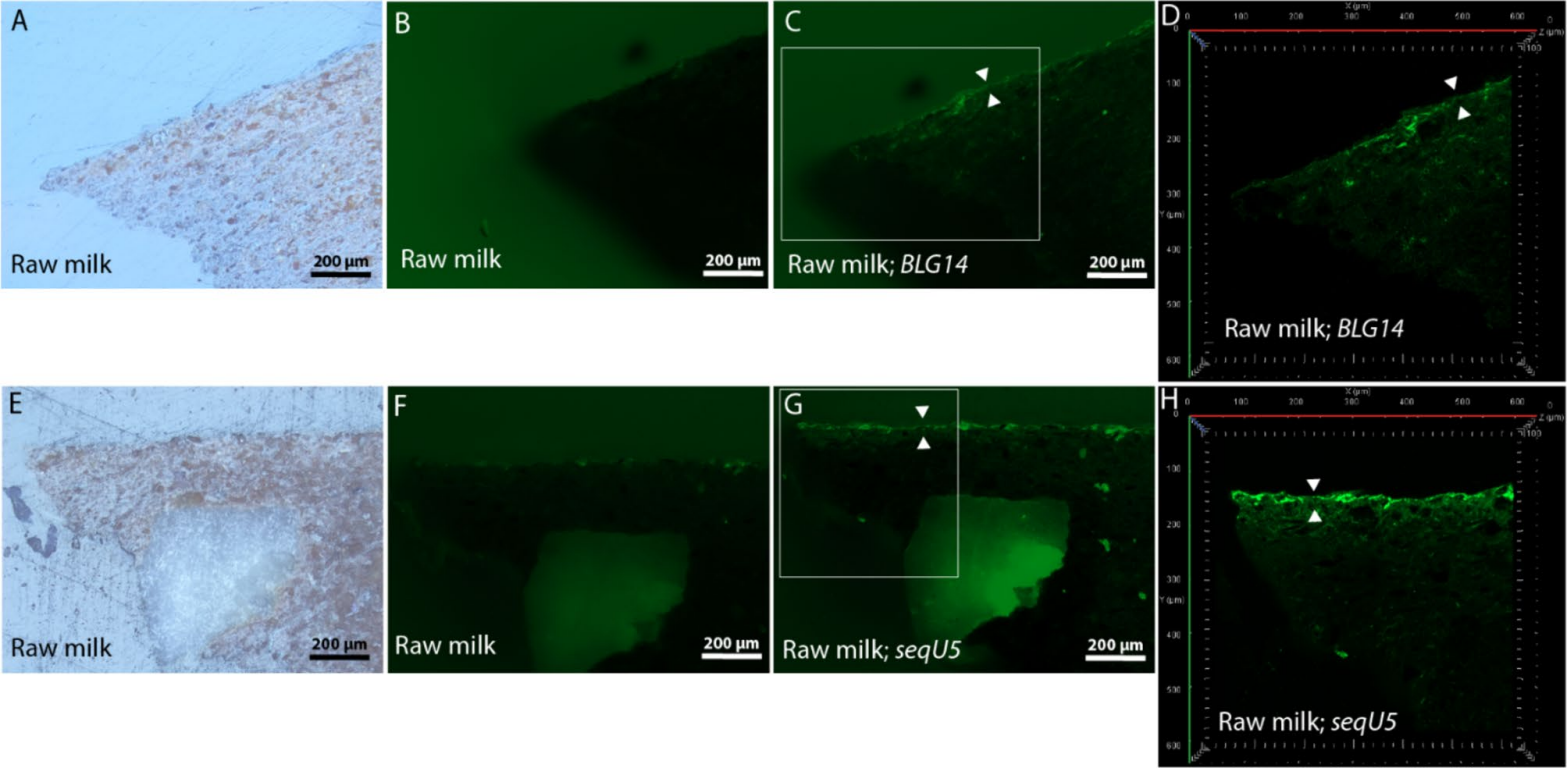
Cross-section images of model samples, which were cooked in Raw milk. They were photographed, prior to hybridisation with the β-lactoglobulin (milk) targeting aptamer *BLG14* (frames C and D) or the β-casomorphin-7 (milk) targeting aptamer *seqU5* (frames G and H), using the reflected-light brightfield observation (frames A and E) and the widefield fluorescence observation (frames B and F) modes. After aptamer hybridisations, samples were again captured in the widefield fluorescence observation mode under the same illumination conditions (frames C and G) and were laser scanned using confocal fluorescence microscopy (frames D and H). Upper layers of ceramic, which were directly exposed to cooking, are marked with white triangles.

Prior to hybridisation, the organic residuals, present within the thin most upper layer of ceramic, which was directly exposed to cooking in raw milk (Fig. 8A and E), exhibited almost no autofluorescence (Fig. 8B and F). After hybridisation with either *BLG14* or *seqU5* aptamers, this layer (defined by white triangles in Fig. 8C andG) was highlighted with fluorescence, which enhanced its brightness under the widefield fluorescence observation mode. These enhancements were even stronger and clearer in the confocal fluorescence observation *mode, in which no material autofluorescence was observed (from resin’s surface reflection and from small stones) (*Fig. 8D and H).

**Figure 9:**
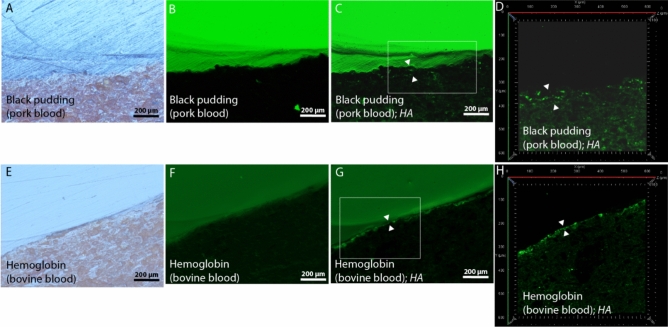
Cross-section images of model samples, which were cooked in a suspension of black pudding or hemoglobin. They were photographed, prior to hybridisation with the hemoglobin (blood) targeting aptamer *HA*, using the reflected-light brightfield observation (frames A and E) and the widefield fluorescence observation (frames B and F) modes. After aptamer hybridisation, samples were again captured in the widefield fluorescence observation mode under the same illumination conditions (frames C and G) and were laser scanned using confocal fluorescence microscopy (frames D and H). Upper layers of ceramic, which were directly exposed to cooking, are marked with white triangles.

Prior to hybridisation, the organic residuals, present within the thin most upper layer of ceramic, which was directly exposed to cooking in a suspension of black pudding or haemoglobin (Fig. 9A and E), exhibited almost no autofluorescence (Fig. 9B and F). After the hybridisation of black pudding or haemoglobin samples with the aptamer HA, this layer (defined by white triangles in Fig. 9C and G) was highlighted with fluorescence, which enhanced its brightness under the widefield fluorescence observation mode (this applies to haemoglobin as well as to black pudding model samples, although the highlighting of the black pudding sample was very slight). These enhancements were stronger and clearer in the confocal fluorescence observation *mode (*Fig. 9H)*, although some unspecific fluorescence within this mode could still be observed (for example on the lower halve of ceramic surface of the* black pudding model sample; Fig. 9D)*.*

**Figure 10:**
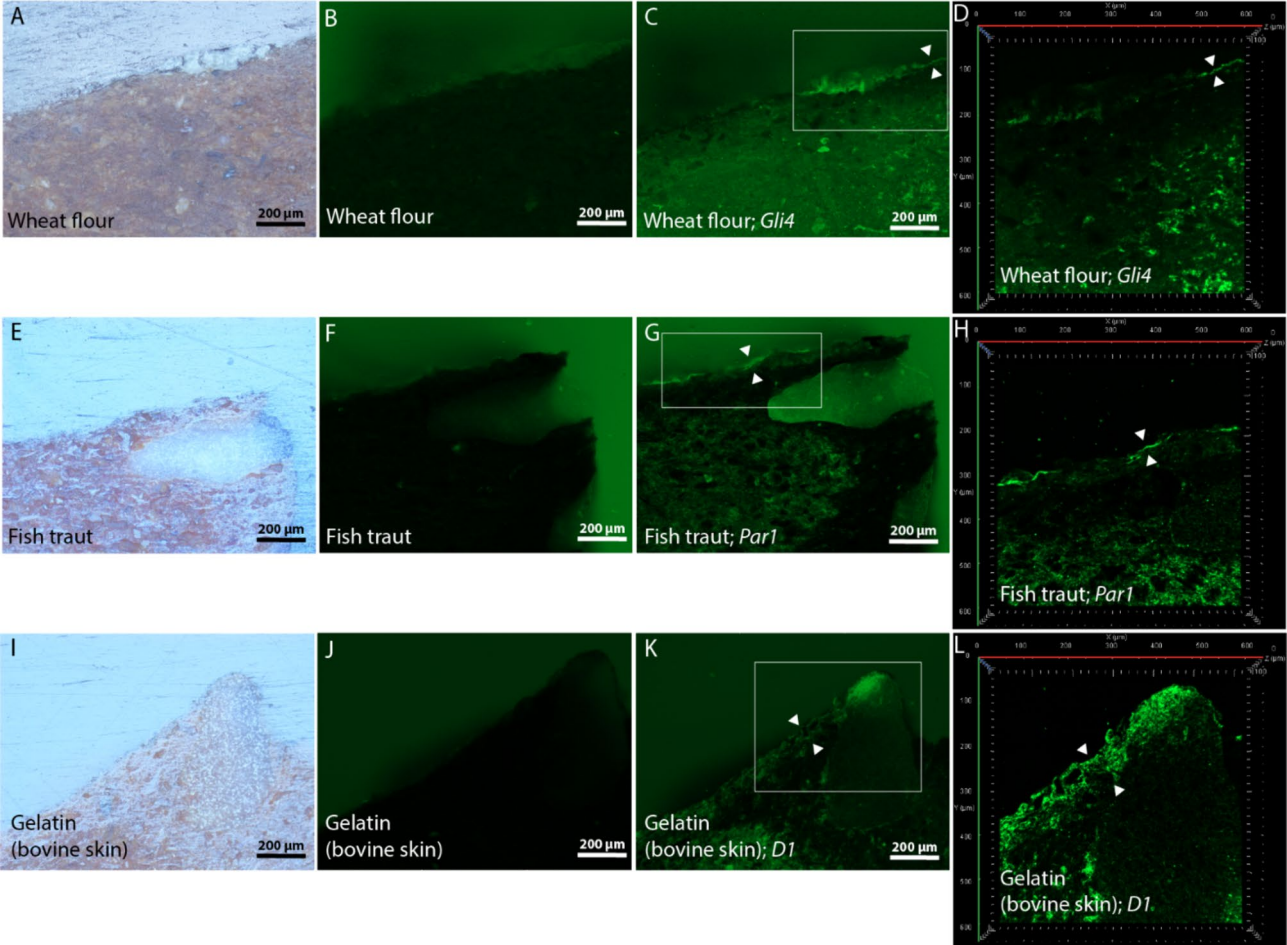
Cross-section images of model samples, which were cooked in a suspension of wheat flour, fish traut or gelatin (bovine skin). They were photographed, prior to hybridisation with the gliadin targeting aptamer *Gli4* (frames C and D) or the parvalbumin (fish) targeting aptamer *Par1* (frames G and H) or with the gelatin aptamer *D1* (frames K and L), using the reflected-light brightfield observation (frames A, E and I) and the widefield fluorescence observation (frames B, F and J) modes. After aptamer hybridisations, samples were again captured in the widefield fluorescence observation mode under the same illumination conditions (frames C, G and K) and were laser scanned using confocal fluorescence microscopy (frames D, H and L). Upper layers of ceramic, which were directly exposed to cooking, are marked with white triangles.

Prior to hybridisation, the organic residuals, present within the thin most upper layer of ceramic, which was directly exposed to cooking in a suspension of wheat flour or fish traut (Fig. 10A and E), exhibited a weak autofluorescence (Fig. 10B and F); and those cooked in a suspension of gelatin (Fig. 10I) had absolutely no autofluorescence (Fig. 10J).

After *Gli4* aptamer hybridisation, a thin layer of ceramic exposed to cooking in a suspension of wheat flour and a whitish non-ceramic layer above it were highlighted with additional fluorescence, which enhanced their brightness under the widefield fluorescence observation mode (Fig. 10C). After *Par1* aptamer hybridisation, a thin layer of ceramic exposed to cooking in a suspension of fish trout, was highlighted with fluorescence (Fig. 10G). Interestingly, in the case of applying the *D1* aptamer, a relatively thick layer of ceramic exposed to cooking in gelatin, was highlighted (Fig. 10K).

For all of the above presented model samples, this highlighting was clearer in the confocal fluorescence observation *mode. Moreover, in the case of* wheat flour, the nonspecific fluorescence of the upper whitish non-ceramic layer, which was observed under the widefield mode, was absent in the confocal *mode (*Fig. 10D*).* Nonetheless, *unspecific fluorescence within the confocal mode could still be observed on the lower halves of ceramic surfaces of* wheat flour and fish traut model samples (Figs. 10H and L)*.*

Immunofluorecence microscopy protocol consists of two washing steps and in most cases, this was sufficeint enough in removing unbound FITC conjugated streptavidin or FITC conjugated secondary antibodies from the polished cross section surfaces. Nevertheless, for two lysozyme model sampels, which were hybridised with eighter *Apt1L* aptamer (Supplementary Fig. 1F) or with anti-ovalbumin Ab (Supplementary Fig. 1L), these two washing steps were insufficient. Consequently, smaller and larger fluorescent stains were scattered around the entirety of these two cross-section images.

## Discussion

Aptamers were assessed for their functionality (specificity) using the ELISA assay. Results indicated that out of the aptamers targeting egg lysozyme, two were functional (*Clone1* and *Kirby*), while one was not (*Apt1L*). For the fish-related material, only aptamers targeting parvalbumin were functional (*Par1*), whereas those targeting histamine did not function (*H2* and *H47*). Additionally, two aptamers targeting haemoglobin showed functionality (*Hb* and *HA*), while one did not (*G15 T1*). Lastly, all aptamers tested against gluten, milk, beef, and collagen demonstrated functionality (*Gli1* and *Gli4* for gluten; *seqU5* and *BLG14* for milk; *Mb1* for beef; and *D1* and *CTx 2R-2 h* for collagen). The ELISA aptamer validation tests showed strong correlation with the results obtained from using aptamers for immunofluorescence microscopy performed on ceramic model samples. Nonetheless, there were four discrepancies between the ELISA and immunofluorescence microscopy results. For example, immunofluorescence microscopy was not successful (after aptamer hybridisation the thin uppermost layer of ceramic, directly exposed to cooking in a protein suspension, did not show bright green fluorescence) when the haemoglobin model sample was hybridized with *Hb*, when the flour model sample was hybridized with *Gli1*, when the beef model sample was hybridized with *Mb1*, and when the gelatin model sample was hybridized with *CTx 2R-2 h*. Therefore, in all of these four cases immunofluorescence microscopy failed even though it was performed using functional aptamers. It is well acknowledged that in ELISA everything is dissolved in liquid and therefore biochemical interactions and reactions work well and just a few molecules of target protein may get detected^48,50,52^. Moreover, the ELISA was performed on freshly prepared protein suspensions, whereas immunofluorescence microscopy was performed on ceramic model samples, which were cooked in an oven for a period of 5 days, UV aged and subsequently buried. Therefore, target proteins may be degraded such that a given epitope may be preferentially lost^48^. Regarding specificity, all ELISA-tested aptamers showed no cross-reactivity with the BSA standard. Additionally, for immunofluorescence microscopy, the hybridization of egg lysozyme targeting *Clone1* or *Apt1L* on ovalbumin model samples, as expected, did not yield a successful immunofluorescence microscopy result.

The functionality of the egg lysozyme targeting aptamer *Kirby* was compared with that of anti-ovalbumin (egg albumin) antibody, which is usually employed for immunofluorescence microscopy for cultural heritage binder analysis^51,53^. After immunofluorescence microscopy on the cross-sections of whole egg control samples, flourescence intensity was 3.1 times greater for the *Kirby* aptamer giving a much clearer picture. Typically, the ordered IgG antibodies (molecular weight ~ 150,000 g/mol) are present in a concentration range of 80–120 μM^54^, while our lyophilized aptamer pellet was diluted to 100 μM. Since both antibody and aptamer stocks were diluted by a factor of 10 before their application onto the cross-section, a similar molecular weight (of antibodies and aptamers) was employed for immunofluorescence microscopy. The greater functionality of aptamers observed here lies in their higher specificity, a result of strict selection during their production involving several SELEX selection cycles. Furthermore, aptamers exhibit high stability at room temperature, a long shelf-life with no batch-to-batch variability, and, compared to antibodies, their molecular weight is only ≈8 kDa, giving them excellent sample penetration^25^.

One thing to note is that during the hybridization of aptamer Kirby or anti-ovalbumin antibody onto the cross-sections of whole egg control samples, the thick egg layer significantly swelled up in size, elevating itself from the rest of the surface. This phenomenon typically occurs when freshly prepared, non-heated and unaged samples are analysed, such as our control samples. Proteins from older materials appear to be sufficiently denatured to become insoluble in aqueous solutions^55^. Moreover, the thicker the proteinaceous layer, the more water it absorbs. Lastly, swelling was not observed in our heated and aged model samples, of which only the thin uppermost layer of ceramic, directly exposed to cooking in a protein suspension, contained residual amounts of protein.

Prior to aptamer hybridisation, the thin uppermost layer of ceramic model samples containing residuals of either egg white, lysozyme, wheat flour or fish traut exhibited a weak autofluorescence. For other model samples no or almost no autofluorescence was observed. In general, proteins contain amino acids tryptophan, tyrosine, and phenylalanine and therefore show some degree of autofluorescence^56^. The weak autofluorescence could therefore reflect the residual presence of proteins within the porous structure of the thin upper ceramic layers of model samples. In any case, autofluorescence did not interfere with the immunofluorescence microscopy results.

The wheat flour model sample, prior to hybridization with *Gli4* (Fig. 10B), showed autofluorescence from a whitish non-ceramic layer on top of the ceramic material. However, this non-ceramic layer was not wheat flour. After excavation, ceramic shards of all model samples were cleaned and rinsed, removing all visual traces of organic residuals (Supplementary Fig. 3). Therefore, this whitish layer resulted from the procedure of embedding the sample in resin, during which an air bubble became entrapped in the solid resin. Subsequently, during the cross-section polishing procedure, a paste of fine solid particles, primarily composed of resin and ceramic, accumulated within this bubble. Most of the paste was removed by eliminating the bubble from the cross-section with further polishing and the use of a needle; however, small fractions still remained.

In comparison to the widefield fluorescence observation mode, the confocal observation mode achieved a much more specific and clear immunofluorescence microscopy picture. This was because in the confocal observation mode nearly all of the unspecific fluorescence originating from micro sample’s autofluorescence (Figs. 10C and D; wheat flour with *Gli4*); from microscopically small stones within the ceramic (Fig. 7G and H; egg white with *Clone1*) or from resin's surface reflection was successfully removed. Nevertheless, the unspecific fluorescence, originating from the lower halves of ceramic surfaces of wheat flour and fish traut model samples, could not have been avoided (Figs. 10D and H). The, confocal observation mode uses a monochromatic laser source to scan across a defined sample area (in z-axis), while the use of a very small aperture (pinhole) in the optical path allows to detect light emitted within the focal plane at different sample depths and, at the same time, to discard the out-of-focus light^52,57^. In this way it is possible to obtain optical sections of the sample and reconstruct its 3D structure from the detected light independent of the position of the scanning spot^50^. This allows for the confocal observation mode to be unaffected by various reflection angles, which form when a complex surface structure, such as a gutter/crack, is exposed to excitation light.

The most capable method for residual protein analysis within ceramics is high-throughput whole protein sequencing using LC–MS/MS proteomics^4,7,28–31,58^. Firstly, proteins need to be extracted from samples requiring at least 10 µg of protein^32^. Regarding the information obtained, immunological methods can hardly compete with proteomics, simply because the obtained amino acid sequences give exact and species-specific results of the food source material for every encountered protein. In immunology, only proteins in question are analysed and not the entire spectrum of extracted proteins (could also be species-specific). Nevertheless, regarding the bottleneck process that is protein extraction (extraction yields of ≤ 0.1%^59^), ELISA, being more sensitive (in theory, it may detect a single positive antigen–antibody binding^60^), requires much lower protein yields compared to proteomics. Moreover, in proteomics, proteins need to be isolated from the extraction buffer (using dialysis or methanol/chloroform procedures), because detergents and urea used in the extraction hinder the very sensitive detection of LC–MS/MS^28^. However, in ELISA, such purifications are unneeded, which means that the sample preparation is faster, retaining more protein for the final analysis^19^. Lastly, in immunofluorescence microscopy, analysis is conducted directly on the sample's cross-section, meaning that protein extraction/purification is entirely excluded^51,53^. Additionally, valuable stratigraphic localization of food proteins on the cross-section can be achieved. This stratigraphic information is lost in proteomics, because samples need to be crushed into ceramic powder prior to protein extraction and purification.

## Conclusions

Our experimental study represents the first application of aptamers as detection molecules in cultural heritage science, aimed at analysing protein residues bound to ceramics. We have successfully validated eight out of sixteen tested aptamers on shards of traditionally prepared, aged, buried (for a year), and excavated archaeological ceramic replicas. These aptamers are designed to detect protein targets originating from various sources, including egg residuals (*Clone1* and *Kirby*), cereal residuals (*Gli4*), milk residuals (*seqU5* and *BLG14*), blood residuals (*HA*), fish residuals (*Par1*), and bone broth residuals (*D1*). Finally, our findings reveal that the specific fluorescence intensities of the aptamer assembly are three times greater than those of standardly employed antibodies, underscoring the superiority of aptamers in this application. Nonetheless, it has to be stated that this is an experimental study conducted exclusively on heated and aged model samples and therefore, for future experimentation, real archaeological case-study samples (at least 100 years old) will have to be included.

### Supplementary Information

Supplementary Information 1.

Supplementary Information 2.

Supplementary Information 3.

Supplementary Information 4.

## Data Availability

All data generated or analysed during this study are included in this published article [and its supplementary information files].

## References

[1] Craig, O. E. *et al.* Earliest evidence for the use of pottery. *Nat. 2013 4967445* **496**, 351–354 (2013). 10.1038/nature12109 23575637

[2] Shelach-Lavi, G. & Tu, D. Food, pots and socio-economic transformation: The beginning and intensification of pottery production in North China. *Archaeol. Res. Asia* **12**, 1–10 (2017). 10.1016/j.ara.2017.10.001

[3] Childe, V. G. Directional changes in funerary practices during 50,000 years. *Man* **45**, 13 (1945). 10.2307/2793007

[4] Hendy, J. Ancient protein analysis in archaeology. *Sci. Adv.* **7**, 9314–9329 (2021). 10.1126/sciadv.abb9314 PMC7810370 33523896

[5] Twiss, K. The archaeology of food and social diversity. *J. Archaeol. Res.* **20**, 357–395 (2012). 10.1007/s10814-012-9058-5

[6] Pleterski, A. Küchenkultur im Frühen Mittelalter. *ZRC SAZU Ljubljana* (2008).

[7] Barker, A. Archaeological protein residues: New data for conservation science. *Ethnobiol. Lett.* **1**, 58–65 (2010). 10.14237/ebl.1.2010.60

[8] Evershed, R. P., Heron, C., Charters, S. & Goad, L. J. The survival of food residues: new methods of analysis, interpretation and application. in *Proceedings of the British Academy* **77**, 187–208 (Publisher: Oxford University Press United Kingdom, 1992).

[9] Regert, M., Garnier, N., Decavallas, O., Cren-Olive, C. & Rolando, C. Structural characterization of lipid constituents from natural substances preserved in archaeological environments. *Meas. Sci. Technol.* **14**, 1620–1630 (2003). 10.1088/0957-0233/14/9/313

[10] Cramp, L. J. *et al.* Lipids in Archaeology. In *Handbook of Archaeological Sciences* (eds Mark Pollard, A. *et al.*) 529–556 (Wiley, 2023). 10.1002/9781119592112.ch26.

[11] Drieu, L. *et al.* Relationships between lipid profiles and use of ethnographic pottery: An exploratory study. *J. Archaeol. Method Theory* **29**, 1294–1322 (2022). 10.1007/s10816-021-09547-1

[12] Cubas, M. *et al.* Latitudinal gradient in dairy production with the introduction of farming in Atlantic Europe. *Nat. Commun.* **11**, 2036 (2020). 32341389 10.1038/s41467-020-15907-4 PMC7184739

[13] Cubas, M. *et al.* Exploring pottery use in the Southwestern Atlantic Europe: an approach from the organic residue analysis. (2018).

[14] Cramp, L. J. E. *et al.* Neolithic dairy farming at the extreme of agriculture in northern Europe. *Proc. R. Soc. B Biol. Sci.* **281**, 1791 (2014). 10.1098/rspb.2014.0819 PMC4132672 25080345

[15] Bondetti, M. *et al.* Fruits, fish and the introduction of pottery in the Eastern European plain: Lipid residue analysis of ceramic vessels from Zamostje 2. *Quat. Int.* **541**, 104–114 (2020). 10.1016/j.quaint.2019.05.008

[16] Fernandes, R. *et al.* Reconstruction of prehistoric pottery use from fatty acid carbon isotope signatures using Bayesian inference. *Org. Geochem.* **117**, 31–42 (2018). 10.1016/j.orggeochem.2017.11.014

[17] Cappellini, E. *et al.* A multidisciplinary study of archaeological grape seeds. *Naturwissenschaften* **97**, 205–217 (2010). 20033124 10.1007/s00114-009-0629-3 PMC2812422

[18] Asara, J. M. *et al.* Interpreting sequences from mastodon and T. Rex. *Science* **317**, 1324–1325 (2007). 17823333 10.1126/science.317.5843.1324

[19] Špec, T. *et al.* CIM® monolith chromatography-enhanced ELISA detection of proteins in artists’ paints: Ovalbumin as a case study. *Microchem. J.* **127**, 102–112 (2016). 10.1016/j.microc.2016.02.004

[20] Craig, O. E. The development of dairying in Europe: potential evidence from food residues on ceramics. *Doc. Praehist.* **29**, 97–107 (2002). 10.4312/dp.29.8

[21] Craig, O. E. & Collins, M. J. The removal of protein from mineral surfaces: Implications for residue analysis of archaeological materials. *J. Archaeol. Sci.* **29**, 1077–1082 (2002). 10.1006/jasc.2001.0757

[22] Craig, O. E. & Collins, M. J. An improved method for the immunological detection of mineral bound protein using hydrofluoric acid and direct capture. *J. Immunol. Methods* **236**, 89–97 (2000). 10699582 10.1016/S0022-1759(99)00242-2

[23] Craig, O. *et al.* Detecting milk proteins in ancient pots. *Nature* **408**, 312 (2000). 11099030 10.1038/35042684

[24] Craig, O. E., Taylor, G., Mulville, J., Collins, M. J. & Pearson, M. P. The identification of prehistoric dairying activities in the Western Isles of Scotland: An integrated biomolecular approach. *J. Archaeol. Sci.* **32**, 91–103 (2005). 10.1016/j.jas.2004.06.009

[25] Zhang, Y., Lai, B. S. & Juhas, M. Recent advances in aptamer discovery and applications. *Molecules* **24**(5), 941 (2019). 30866536 10.3390/molecules24050941 PMC6429292

[26] Nimjee, S. M., White, R. R., Becker, R. C. & Sullenger, B. A. Aptamers as Therapeutics. *Annu. Rev. Pharmacol. Toxicol.* **57**, 61–79 (2017). 28061688 10.1146/annurev-pharmtox-010716-104558 PMC6035745

[27] Hennicke, H. W. & Hesse, A. Traditional ceramics. in *Concise Encyclopedia of Advanced Ceramic Materials* 488–494 (Elsevier, 1991).

[28] Barker, A. *et al.* An optimized approach for protein residue extraction and identification from ceramics after cooking. *J. Archaeol. Method Theor.* **19**(3), 407–439 (2012). 10.1007/s10816-011-9120-5

[29] Dallongeville, S. *et al.* Dealing with the identification of protein species in ancient amphorae. *Anal. Bioanal. Chem.* **399**, 3053–3063 (2011). 20890751 10.1007/s00216-010-4218-2

[30] Pal Chowdhury, M., Makarewicz, C., Piezonka, H. & Buckley, M. Novel deep eutectic solvent-based protein extraction method for pottery residues and archeological implications. *J. Proteome Res.* **21**, 2619–2634 (2022). 36268809 10.1021/acs.jproteome.2c00340 PMC9639204

[31] Solazzo, C., Fitzhugh, W. W., Rolando, C. & Tokarski, C. Identification of protein remains in archaeological potsherds by proteomics. *Anal. Chem.* **80**, 4590–4597 (2008). 18494502 10.1021/ac800515v

[32] Hendy, J. *et al.* Ancient proteins from ceramic vessels at Çatalhöyük West reveal the hidden cuisine of early farmers. *Nat. Commun.*10.1038/s41467-018-06335-6 (2018). 30283003 10.1038/s41467-018-06335-6 PMC6170438

[33] Cox, J. C. & Ellington, A. D. Automated selection of anti-protein aptamers. *Bioorganic Med. Chem.* **9**, 2525–2531 (2001). 10.1016/S0968-0896(01)00028-1 11557339

[34] Kirby, R. *et al.* Aptamer-based sensor arrays for the detection and quantitation of proteins. *Anal. Chem.* **76**, 4066–4075 (2004). 15253644 10.1021/ac049858n

[35] Tran, D. T. *et al.* Selection and characterization of DNA aptamers for egg white lysozyme. *Mol.* **15**, 1127–1140 (2010). 10.3390/molecules15031127 PMC6257241 20335968

[36] Amaya-González, S., de Los-Santos-Álvarez, N., Miranda-Ordieres, A. J. & Lobo-Castañón, M. J. Sensitive gluten determination in gluten-free foods by an electrochemical aptamer-based assay. *Anal. Bioanal. Chem.* **407**, 6021–6029 (2015). 26048055 10.1007/s00216-015-8771-6

[37] Parashar, A., Rajput, Y. S. & Sharma, R. Aptamer-based sensing of β-casomorphin-7. *J. Agric. Food Chem.* **63**, 2647–2653 (2015). 25712869 10.1021/acs.jafc.5b00007

[38] Eissa, S. & Zourob, M. In vitro selection of DNA aptamers targeting β-lactoglobulin and their integration in graphene-based biosensor for the detection of milk allergen. *Biosens. Bioelectron.* **91**, 169–174 (2017). 28006685 10.1016/j.bios.2016.12.020

[39] Almusharraf, A. Y., Eissa, S. & Zourob, M. Truncated aptamers for total and glycated hemoglobin, and their integration into a graphene oxide-based fluorometric method for high-throughput screening for diabetes. *Mikrochim. Acta*10.1007/s00604-018-2789-3 (2018). 29675559 10.1007/s00604-018-2789-3

[40] Duanghathaipornsuk, S. *et al.* Aptamer-embedded DNA origami cage for detecting (glycated) hemoglobin with a surface plasmon resonance sensor. *Mater. Lett.* **275**, 128141 (2020). 10.1016/j.matlet.2020.128141

[41] Lin, H. I. *et al.* Selection of aptamers specific for glycated hemoglobin and total hemoglobin using on-chip SELEX. *Lab Chip* **15**, 486–494 (2015). 25408102 10.1039/C4LC01124D

[42] Sun, C. *et al.* One-step green synthesis of a polypyrrole-Au nanocomposite and its application in myoglobin aptasensor. *Anal. Methods* **7**, 5262–5268 (2015). 10.1039/C5AY01006C

[43] Wang, Y., Li, H., Zhou, J., Qi, Q. & Fu, L. A colorimetric and fluorescent gold nanoparticle-based dual-mode aptasensor for parvalbumin detection. *Microchem. J.* **159**, 105413 (2020). 10.1016/j.microc.2020.105413

[44] Lerga, T. M. *et al.* Gold nanoparticle aptamer assay for the determination of histamine in foodstuffs. *Microchim. Acta*10.1007/s00604-020-04414-4 (2020). 10.1007/s00604-020-04414-4 32676707

[45] John Ho, L. S., Fogel, R. & Limson, J. L. Generation and screening of histamine-specific aptamers for application in a novel impedimetric aptamer-based sensor. *Talanta* **208**, 120474 (2020). 31816738 10.1016/j.talanta.2019.120474

[46] Lorenzo-Gómez, R., Miranda-Castro, R., de Los Toyos, J. R., De Los-Santos-Álvarez, N. & Lobo-Castañón, M. J. Aptamers targeting a tumor-associated extracellular matrix component: The human mature collagen XIα1. *Anal. Chim. Acta* **1189**, 339206 (2022). 34815029 10.1016/j.aca.2021.339206

[47] Bruno, J. G., Carrillo, M. P., Phillips, T., Hanson, D. & Bohmann, J. A. DNA aptamer beacon assay for C-telopeptide and handheld fluorometer to monitor bone resorption. *J. Fluoresc.* **21**, 2021–2033 (2011). 21643742 10.1007/s10895-011-0903-6

[48] Heginbotham, A., Millay, V. & Quick, M. The Use of Immunofluorescence Microscopy and Enzyme-Linked Immunosorbent Assayas Complementary Techniques for Protein Identification in Artists’ Materials. *J. Am. Inst. Conserv.* **45**, 89–105 (2006). 10.1179/019713606806112522

[49] Petty, H. R. Fluorescence microscopy: Established and emerging methods, experimental strategies, and applications in immunology. *Microsc. Res. Tech.* **70**, 687–709 (2007). 17393476 10.1002/jemt.20455

[50] Cartechini, L., Palmieri, M., Vagnini, M. & Pitzurra, L. Immunochemical methods applied to art-historical materials: Identification and localization of proteins by ELISA and IFM. *Top. Curr. Chem.* **374**, 1–21 (2016). 10.1007/s41061-015-0006-y 27572988

[51] Kosel, J., Tavzes, Č, Retko, K. & Ropret, P. Can traditional artist’s pigments hinder paint binder characterization using immunofluorescence microscopy? Application of widefield fluorescent and confocal laser scanning microscopies for advanced imaging and surface topography scans. *J. Cult. Herit.* **61**, 76–90 (2023). 10.1016/j.culher.2023.03.006

[52] Cartechini, L. *et al.* Immunodetection of proteins in ancient paint media. *Acc. Chem. Res.* **43**, 867–876 (2010). 20438070 10.1021/ar900279d

[53] Kosel, J., Kavkler, K., Pološki, N. & Ropret, P. Immunofluorescence microscopy for the characterization of paint binder in wall paintings: A two-step procedure of using anti-ovalbumin and anti-casein antibodies on the same micro sample. *J. Cult. Herit.* **66**, 271–281 (2024). 10.1016/j.culher.2023.12.001

[54] Jazayeri, M. H., Pourfathollah, A. A., Rasaee, M. J., Porpak, Z. & Jafari, M. E. The concentration of total serum IgG and IgM in sera of healthy individuals varies at different age intervals. *Biomed. Aging Pathol.* **3**, 241–245 (2013). 10.1016/j.biomag.2013.09.002

[55] Kockaert, L., Gausset, P. & Dubi-Rucquoy, M. Detection of ovalbumin in paint media by immunofluorescence. *Stud. Conserv.* **34**, 183–188 (1989). 10.1179/sic.1989.34.4.183

[56] Verma, R. *et al.* Detection and identification of amino acids and proteins using their intrinsic fluorescence in the visible light spectrum. *Anal. Chim. Acta* **1282**, 341925 (2023). 37923411 10.1016/j.aca.2023.341925

[57] Pawley, J. B. Fundamental Limits in Confocal Microscopy. *Handb. Biol. Confocal Microsc. Third Ed.* 20–42 (2006). 10.1007/978-0-387-45524-2_2

[58] Barker, A. *et al.* Validaton of a non-targeted LC-MS approach for identfying ancient proteins: Method development on bone to improve artfact residue analysis. *Ethnobiol. Lett.* **6**, 162–174 (2015). 10.14237/ebl.6.1.2015.294

[59] Ren, F., Atlasevich, N., Baade, B., Loike, J. & Arslanoglu, J. Influence of pigments and protein aging on protein identification in historically representative casein-based paints using enzyme-linked immunosorbent assay. *Anal. Bioanal. Chem.* **408**, 203–215 (2016). 26472321 10.1007/s00216-015-9089-0

[60] Chang, L. *et al.* Single molecule enzyme-linked immunosorbent assays: Theoretical considerations. *J. Immunol. Methods* **378**, 102 (2012). 22370429 10.1016/j.jim.2012.02.011 PMC3327511

