# Supplementary figures and images for: The potential of aptamers for the analysis of ceramic bound proteins found within pottery
Source: Sci Rep. 2024 Aug 27;14:19947. doi: 10.1038/s41598-024-70048-8 (PMC11358422; doi:10.1038/s41598-024-70048-8)

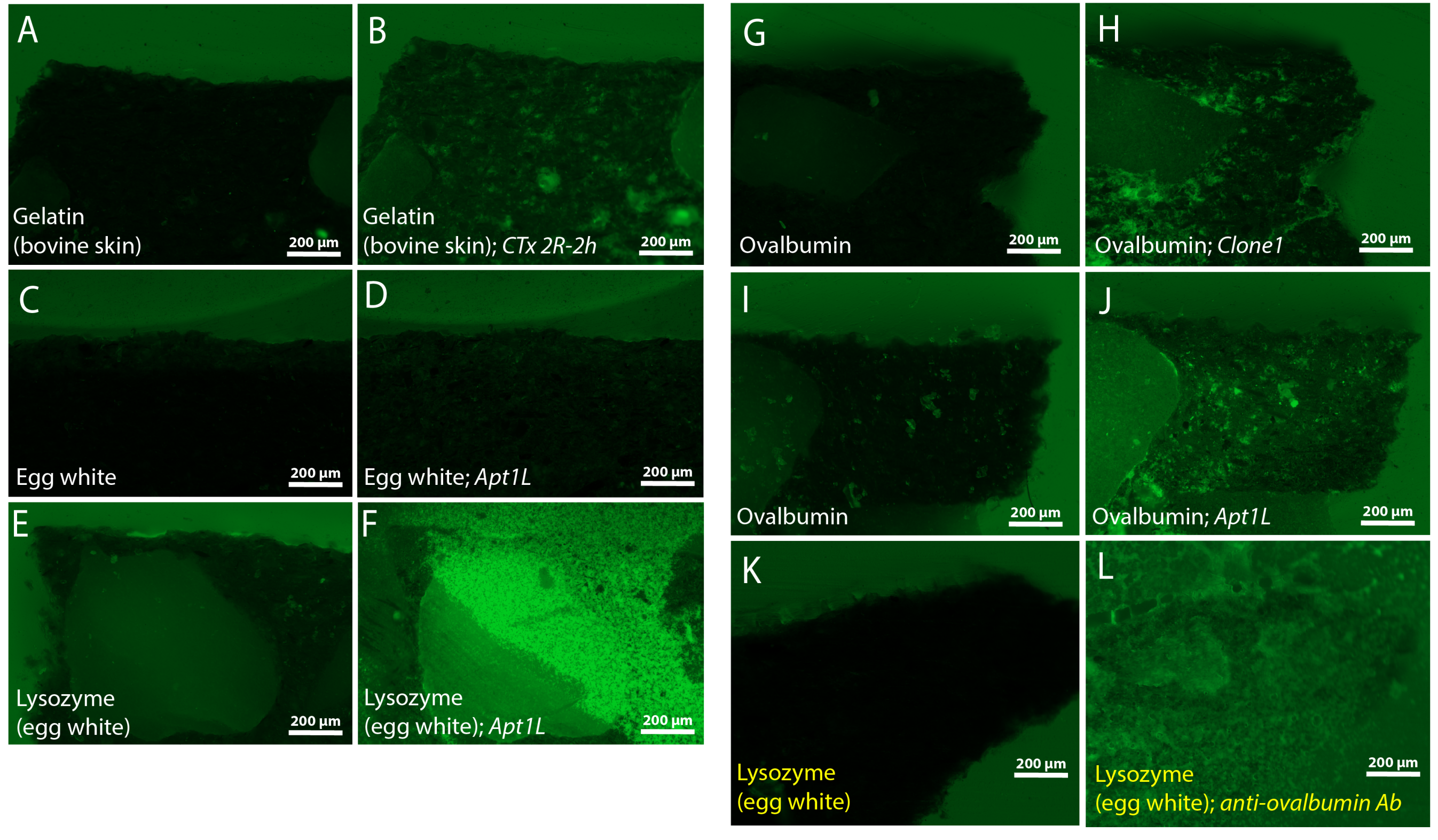

Supplement: Supplementary file 1 — Supplementary Information 1. [file 41598_2024_70048_MOESM1_ESM.tif]

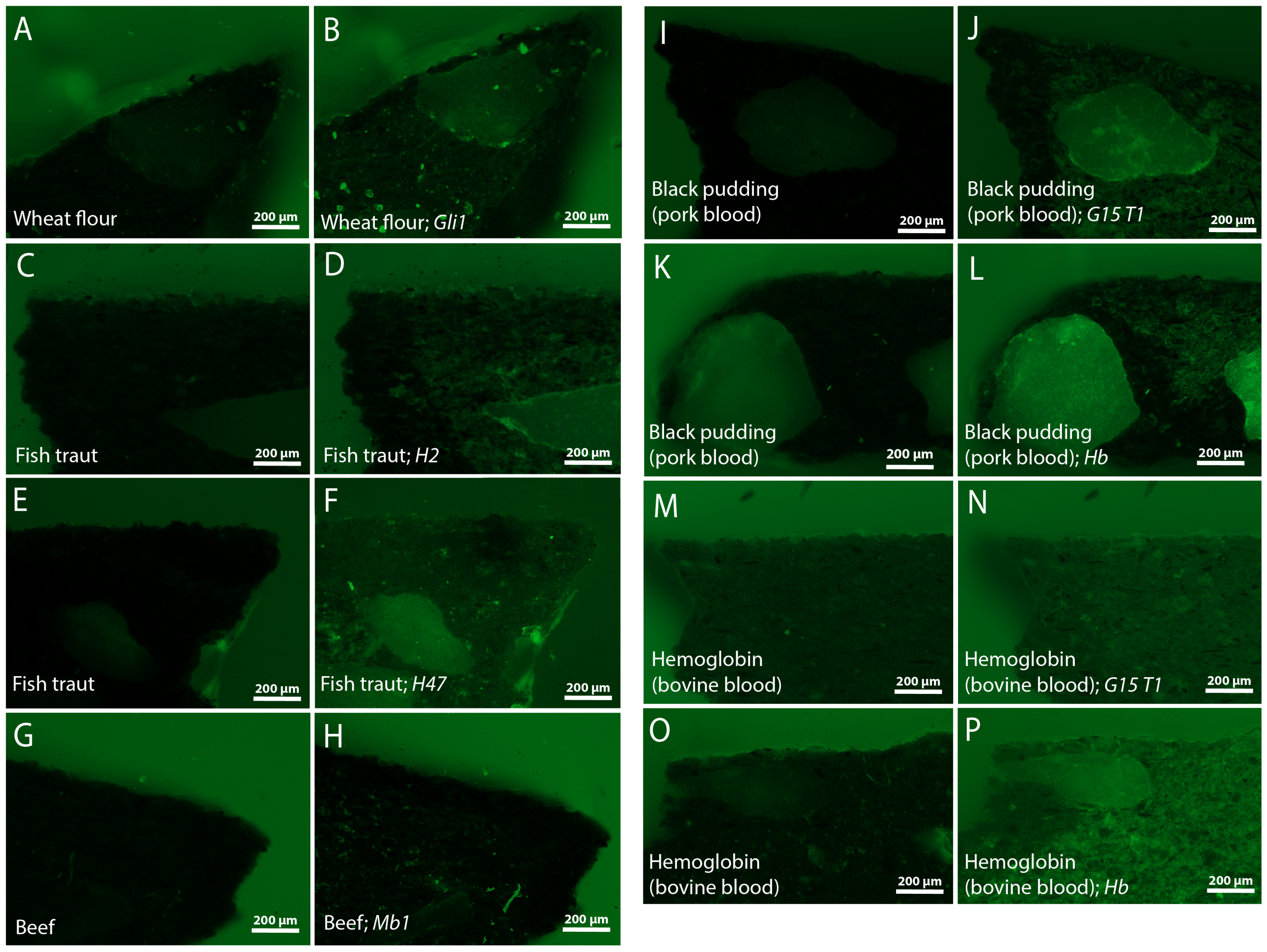

Supplement: Supplementary file 2 — Supplementary Information 2. [file 41598_2024_70048_MOESM2_ESM.tif]

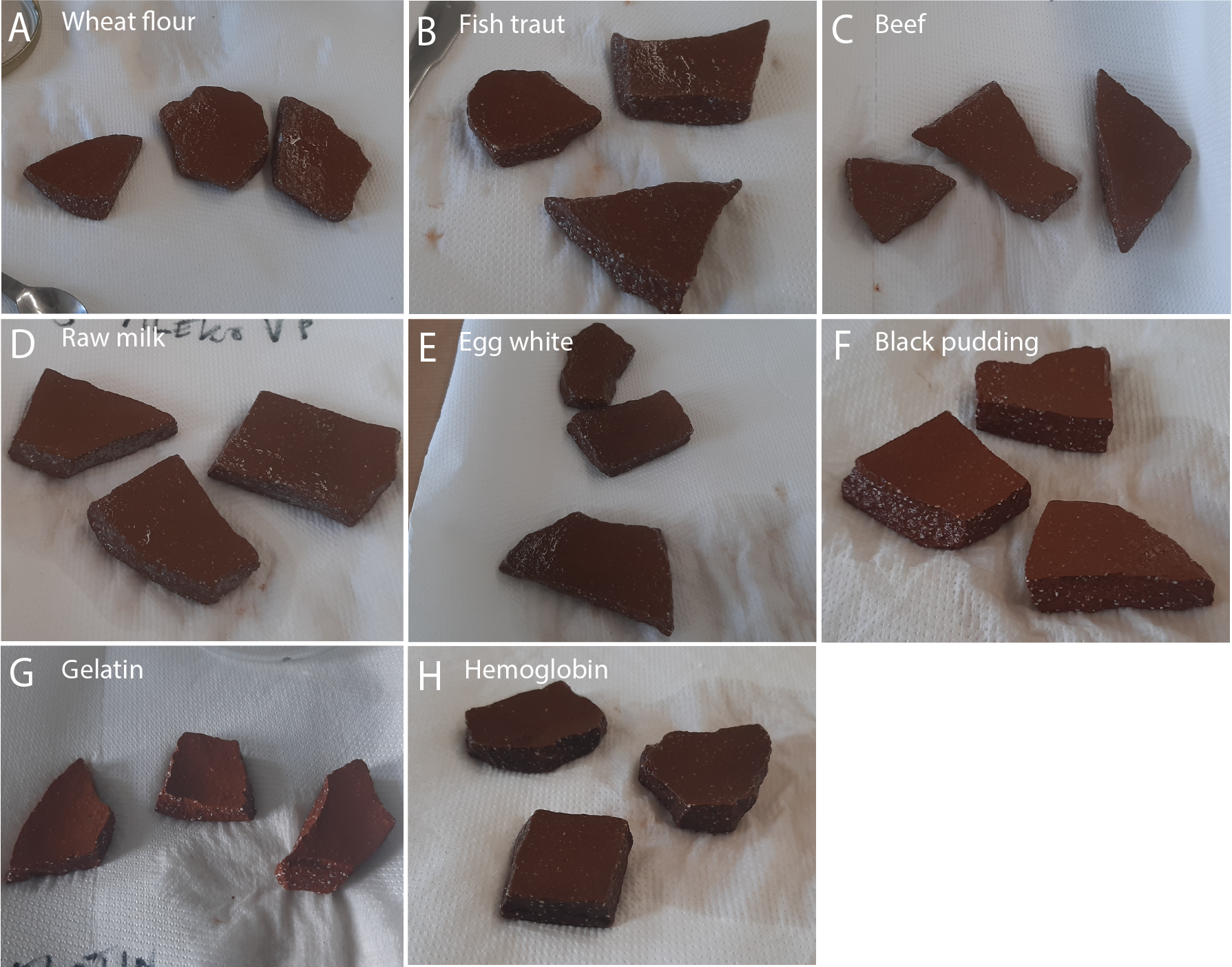

Supplement: Supplementary file 3 — Supplementary Information 3. [file 41598_2024_70048_MOESM3_ESM.tif]
