# Supplementary material for: The potential of aptamers for the analysis of ceramic bound proteins found within pottery
Source: Sci Rep. 2024 Aug 27;14:19947. doi: 10.1038/s41598-024-70048-8 (PMC11358422; doi:10.1038/s41598-024-70048-8)
Supplement: Supplementary file 4 — Supplementary Information 4. [file 41598_2024_70048_MOESM4_ESM.docx]

Supplementary Table 1: Testing of sixteen aptamers using the ELISA assay. Presented values are measurements of OD_405nm_. Each OD_405nm_ value has a standard error assigned to it.

|  |  |  |  |  |  |  |
| --- | --- | --- | --- | --- | --- | --- |
|  | **Trget protein** | **Designation** | **ELISA (OD_405nm_)** | | |  |
|  |  |  | **MilliQ** | **BSA** | **Target protein** |  |
|  | Lysozyme | Clone1 | 0.034 ± 0.009 | 0.027 ± 0.007 | 0.463 ± 0.087 |  |
|  |  | Kirby | 0.010 ± 0.005 | 0.024 ± 0.009 | 0.380 ± 0.045 |  |
|  |  | Apt1L | 0.048 ± 0.015 | 0.020 ± 0.017 | 0.037 ± 0.011 |  |
|  | Gluten | Gli 4 | 0.012 ± 0.003 | 0.078 ± 0.046 | 0.519 ± 0.052 |  |
|  |  | Gli 1 | 0.041 ± 0.018 | 0.073 ± 0.013 | 0.577 ± 0.069 |  |
|  | Casein | seqU5 | 0.065 ± 0.016 | 0.004 ± 0.006 | 0.510 ± 0.062 |  |
|  | β-Lactoglobulin | BLG14 | 0.054 ± 0.019 | 0.023 ± 0.012 | 0.394 ± 0.022 |  |
|  | Hemoglobin | G15 T1 | 0.013 ± 0.003 | 0.008 ± 0.002 | 0.015 ± 0.010 |  |
|  |  | HA | 0.018 ± 0.011 | 0.062 ± 0.025 | 0.471 ± 0.153 |  |
|  |  | Hb | 0.071 ± 0.026 | 0.082 ± 0.037 | 0.388 ± 0.056 |  |
|  | Myoglobin | Mb1 | 0.044 ± 0.017 | 0.024 ± 0.013 | 0.488 ± 0.063 |  |
|  | Parvalbumin | Par1 | 0.018 ± 0.006 | 0.061 ± 0.022 | 0.412 ± 0.093 |  |
|  | Histamine | H2 | 0.046 ± 0.004 | 0.014 ± 0.003 | 0.087 ± 0.033 |  |
|  |  | H47 | 0.023 ± 0.011 | 0.029 ± 0.024 | 0.031 ± 0.012 |  |
|  | Collagen | D1 | 0.034 ± 0.009 | 0.073 ± 0.041 | 0.400 ± 0.045 |  |
|  |  | CTx 2R-2h | 0.023 ± 0.015 | 0.061 ± 0.028 | 0.615 ± 0.142 |  |
|  |  |  |  |  |  |  |
